# Defining skin aging and its risk factors: a systematic review and meta-analysis

**DOI:** 10.1038/s41598-021-01573-z

**Published:** 2021-11-11

**Authors:** Qi Yi Ambrose Wong, Fook Tim Chew

**Affiliations:** Allergy and Molecular Immunology Laboratory, Lee Hiok Kwee Functional Genomics Laboratories, Department of Biological Sciences, Faculty of Science, National University of Singapore, Block S2, Level 5, 14 Science Drive 4, Lower Kent Ridge Road, Singapore, 117543 Singapore

**Keywords:** Risk factors, Skin manifestations

## Abstract

Skin aging has been defined to encompass both intrinsic and extrinsic aging, with extrinsic aging effected by environmental influences and overlaying the effects of chronological aging. The risk factors of skin aging have been studied previously, using methods of quantifying skin aging. However, these studies have yet to be reviewed. To better understand skin aging risk factors and collate the available data, we aimed to conduct a systematic review and meta-analysis. We conducted our systematic review in compliance with Preferred Reporting Item for Systematic Review and Meta-Analyses (PRISMA) guidelines. Embase, PubMed and Web of Science databases were searched in October 2020 using specific search strategies. Where odds ratios were reported, meta-analyses were conducted using the random effects model. Otherwise, significant factors were reported in this review. We identified seven notable risk factors for various skin aging phenotypes: age, gender, ethnicity, air pollution, nutrition, smoking, sun exposure. This review’s results will guide future works, such as those aiming to examine the interaction between genetic and environmental influences.

## Introduction

### Background

Epidemiological evidence of environmentally induced skin changes has existed as early as 1965^1^. However, the concept of skin aging as a superimposition of skin changes induced by both chronologic and environmental factors was only introduced later, after 1983^2,3^. Yet, a formally agreed definition of skin aging and its signs is still lacking. There is a rough consensus that skin aging encompasses several phenotypes such as, but not limited to, wrinkling, pigmentation and telangiectasis^3–8^. As such, skin aging has been quantified using different phenotypes and grading systems, allowing the identification of multiple risk factors by various epidemiological studies^9,10^.

### Definition of skin aging

In this article, skin aging is defined simply as changes to the skin that occur due to aging. Attention was paid to the phrases ‘changes to the skin’, and ‘aging’. With reference to the former, changes to the skin may be categorised as, but not limited to the following: histological, morphological, and physiological. The latter phrase, ‘aging’, requires careful elucidation. Forming an interface between the human body and the external environment, the human skin is constantly subject to both chronologically and environmentally induced changes. Thus, skin aging may be categorised as intrinsic or extrinsic, depending on the epidemiological factors affecting the skin aging process, whereby intrinsic aging is attributed chronological and genetic factors, while extrinsic aging is influenced by environmental factors^11^.

### Aim of review

This review examines the association of epidemiological factors with human skin aging signs that are assessable by non-invasive means. We aimed to obtain a broad overview of the epidemiology of skin aging; hence, all participant subjects available in the literature were considered. Although the focus was on modifiable epidemiological factors (extrinsic aging), intrinsic aging factors were included due to their potential interaction or confounding effects with extrinsic factors. The outcome of interest was skin aging signs assessable through non-invasive means, with emphasis on visual assessment methods, owing to the convenience of execution and cosmetic implications of visually evident skin aging phenotypes. Lastly, to maximise the scope of possible associations, we focused on non-experimental observational studies (i.e., cross-sectional, or longitudinal study designs).

## Methodology

### Search strategy

**Figure 1:**
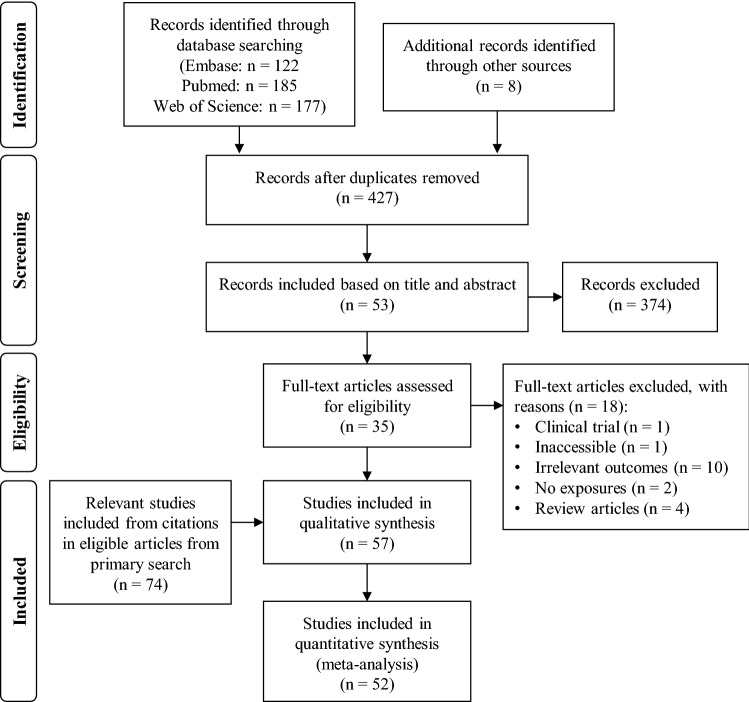
PRISMA flow diagram (from Moher et al.^12^. Preferred Reporting Items for Systematic Reviews and Meta-Analyses: The PRISMA Statement. PLoS Med 6(7): e1000097. https://doi.org/10.1371/journal.pmed1000097).

This review was conducted in accordance with the Preferred Reporting Item for Systematic Review and Meta-Analyses (PRISMA) guidelines^12^ (see Supplementary Table 1 for PRISMA checklist). A primary literature search was performed using the Embase, PubMed and Web of Science databases in October 2020. Search results were restricted to English journal articles published between 1990 and 2020. The search term for all databases included ‘skin aging’ or ‘skin ageing’ in the title or abstract, and ‘risk’, or ‘protective’, or ‘epidemiology’ in all index fields. Full search terms and filters applied for their respective databases are summarised in Table 1. Eligible articles from the primary search were determined using pre-defined eligibility criteria. A secondary search was conducted by hand-searching references cited by the eligible articles from the primary search. Results obtained in the secondary search were deduplicated and screened using the same eligibility criteria as that applied in the primary search. The hand-search process was repeated for results from the secondary search to ensure a thorough record was obtained (see Fig. 1 for PRISMA flow diagram). A final search, wherein a list of phenotypes collected from the results of the literature search were used as keywords, only returned records that were duplicates of previous results or irrelevant to this review’s aims, ensuring that the literature search illustrated in Fig. 1 was sufficiently thorough.

**Table 1:** Full search terms and filters applied for their respective databases during the literature search.

Database	Full search term	Filters applied
Embase	(Skin NEAR/5 (aging OR ageing)):ab,ti) AND (risk OR protective OR epidemiology)	Article, article in press
		English
		Epidemiology
		Etiology
		Prevention
Pubmed	(Skin ageing[Title/Abstract]) AND (risk OR protective OR epidemiology)	Journal article
		English
Web of science	TS = (skin NEAR/5 ag*ing) AND ALL = (risk OR protective OR epidemiology)	Article
		English
		Dermatology

### Eligibility criteria

As per our definition of skin aging and stated aims, articles eligible for inclusion in this review examined associations of epidemiological factors, both intrinsic and extrinsic, with skin aging outcomes in any human subjects assessed via non-invasive methods, by means of a non-experimental observational study. Any full-text journal article available in English was included. Articles excluded from the review for met at least one of the following criteria: study pertained to non-human subjects (e.g., in-vitro studies, murine experiments); study did not examine any epidemiological exposure factors; epidemiological exposures examined by study were disease or disorders (e.g., cancers, eczema); study examined skin aging signs as risk factors for other clinical conditions; study pertained to non-epidemiological risk factors (e.g., genetic polymorphisms); study quantified skin aging via invasive methods (e.g., skin biopsy) or instrumental skin parameter measurements (e.g., skin pH, trans-epidermal water loss); article focussed on clinical trials. Several articles reported outcomes measured using various methods (invasive, non-invasive, or instrumental measurement); if outcomes obtained via non-invasive assessment methods were reported, the study was included. Outcomes quantified using digital image analysis were also included. Using this eligibility criteria, a total of 490 records were screened. An initial screening was first performed by selecting articles based on their title and abstract, before retrieving full-text reports for selected articles and re-assessing them for suitability. Full-text articles excluded from the review met at least one of the following criteria: article was a review; article reported insufficient study data; article reported only analysed correlations between skin aging signs. Additionally, quality assessments of included studies were performed using JBI critical appraisal tools^13^.

### Data extraction

The following study characteristics were abstracted from the full-text articles: author(s) and year, country, sample size and subject demographics, outcome details (domain, anatomical location, definition if any, and method of quantification), and exposures (protective or risk factor). In several studies, the number of respondents and number of subject data used in statistical analysis differed; the number of subjects included in statistical analysis, if reported, was extracted, and considered as the sample size. To account for varying terminology used by different studies for identical outcomes, we categorised outcomes into 24 individual domains (e.g., pigmentation, wrinkling), including an ‘other’ category which comprised outcomes unique to its study and for which no clear definition was provided (e.g., tear troughs, oral commissures) (Supplementary Table 2). For quantitative data, the following were extracted: estimates of effect sizes; confidence interval or standard error, whichever was reported. Where possible, adjusted effect sizes were preferred to non-adjusted ones. If effect sizes were not reported, means and error bars were extracted (Supplementary Dataset). This review focussed primarily on odds ratios.

### Statistical analysis

Meta-analyses were conducted on R with the RStudio interface^14^, using the metafor package^15^. Meta-analysis for a given risk factor was conducted when an effect size estimate for association between an outcome of interest and said risk factor was reported by at least two independent studies, using the DerSimonian-Laird random effects meta-analysis method to account for between-study heterogeneity^16^. Pooled odds ratios (pOR) were obtained from the analyses. Odds ratios included in the random effects models were also ensured to compare identical or non-overlapping exposure and reference categories (i.e., subjects grouped in a given exposure category in one study did not qualify to be grouped into the reference category from another study). To avoid possible overlap of study populations and inclusion of duplicate results in the meta-analytic models, each study was only represented once for a given outcome, unless effect sizes reported were for stratified populations. Additionally, it was ensured that each study’s subjects were not drawn from the same study cohort. Where overlapping study subjects were concerned, only the larger study was considered. To assess heterogeneity, the I^2^ index was calculated for each meta-analysis; an I^2^ value upwards of 50% with p-value < 0.05 was considered as a suggestion of significant heterogeneity. Begg’s funnel plots and Egger’s test were used to assess publication bias; Egger’s test was only performed for meta-analysis of more than 2 studies.

## Results and discussion

### Literature search

Thirty-four eligible journal articles were identified during the primary search and screening process (Fig. 1). These studies reported on associations between risk factors and outcomes which they linked to skin aging. However, phenotypes considered as skin aging signs or used as measures of skin aging were found to differ between studies. Thus, literature cited by eligible articles from the initial search were retrieved and screened; this citation screening process constituted a secondary literature search. Eligible articles from this secondary search were subjected to the same citation screening process to obtain additional studies, which when deduplicated and included with the results from the secondary search, yielded a total of 74 eligible articles. These articles examined outcomes linked to skin aging by articles from the primary search and reported risk factor associations but did not necessarily link the outcomes to skin aging themselves. The final database search using outcomes from the eligible primary search results did not yield other eligible studies not already included in this review. This paper reviews 109 studies (Supplementary References). Funnel plots, often asymmetrical, and Egger’s test p-values indicated that there was often significant publication bias across all outcomes (Supplementary Figs. 2–10).

### Overview of study characteristics

Study populations originated from 16 countries (Supplementary Table 2). Sample sizes and subject characteristics varied between studies, with samples sizes ranging from 24 females only^17^, to 20,295 males and females from the NHANES study^18^. Some studies examined specific races or genders, while others were nationwide cohort studies (e.g., NHANES). The narrowest sample age range reported was 13–15 years^19^ and the widest was 18–96 years^20^, with the youngest subject aged one^18^ and the oldest aged 101 years^21^.

### Overview of skin aging definitions

Twenty-nine articles offered some definition of skin aging, with 11 from the primary search and 18 from the secondary search (Supplementary Table 3). Conversely, articles with no clear definition of skin aging generally listed skin aging signs attributable to skin aging, or factors influencing skin aging. Although this review did not identify a formal definition of human skin aging in the literature, the rough consensus in included articles was to attribute human skin aging to both intrinsic aging and aging influenced by external factors. Intrinsic skin aging was attributed to non-modifiable risk factors such as chronological aging and genetic influences, while extrinsic aging was attributed to modifiable extrinsic factors, such as sun exposure and smoking. From the primary search, there were instances of the terms ‘photoaging’ being used in place of or equated to ‘extrinsic aging’^22–25^, while others defined extrinsic aging as skin aging influenced by multiple extrinsic risk factors, including skin aging attributed to sun or ultraviolet (UV) exposure which ‘photoaging’ refers to, but not vice versa. This review considers photoaging as a proper subset of extrinsic aging. The visible skin aging phenotype is thus the superimposition of intrinsic and extrinsic skin aging signs—a point that was acknowledged and accounted for by at least two validated skin aging scores that relied on visual grading of outcomes^7,11^.

Considering the lack of a standardised definition of skin aging, definitions provided in reviewed studies, and the content of the foregoing studies, we propose the following definition for skin aging: “Skin aging is a superimposition of benign skin phenotypes indicative of histological and morphological changes which are both continuous and inevitable, caused by both intrinsic and extrinsic factors, wherein genetic and chronological influences constitute the former, and environmental influences constitute the latter.” With this definition, we aim to succinctly encompass the relevant definitions identified in the literature while progressing towards a standardised definition of skin aging.

### Overview of skin aging signs and non-invasive assessment methods

Fifty-one studies linked their study outcome or outcomes to skin aging, from which 24 skin aging outcomes were identified. These studies examined a variety of phenotypes, the most frequent of which were pigmentation (n = 17), sagging (n = 12), telangiectasia (n = 11), and wrinkling (n = 35). Notably, the term pigmentation often referred to changes in pigmentation, dyspigmentation, or the formation of pigment spots; we henceforth refer to this phenotype as dyspigmentation. Additionally, we observed repeated usage of the Beagley-Gibson photonumeric scale for skin surface microtopography (SSM), wherein a higher score indicated a greater degree of skin aging^26^. Skin aging was also assessed as an outcome comprising a collection of phenotypes (n = 7): overall skin aging using SCINEXA^27^, perceived age^28,29^, or photoaging^20,30–32^. Overall, most methods of outcome quantification entailed visual assessment by clinicians or researchers according to arbitrary grading scales (Supplementary Table 1), such as the Daniell wrinkling scale^33^ and the Beagley-Gibson grading scale for SSM^26^, both of which were used recurrently in different studies. Studies used grading scales (n = 68) which were often ordinal wherein higher score values indicated greater severity of skin aging outcome. Text definitions, photographs, or both, were provided as scale references and served as a direct measure of target outcomes. In contrast, three studies assigned grades to their target outcome, then calculated a final score from the assigned grades by applying a formula^34–36^. For discrete outcomes (e.g., seborrheic keratosis), studies assessed subjects by determining presence or absence (n = 14), counting the number of occurrences (n = 10), or both counting, then assigning a grade according to the frequency of outcome (n = 8). Other studies assessed the global face by estimating perceived age (n = 5) or conducted digital image analyses (n = 19) which offered a more precise and objective measure of outcome (e.g., measurement of total wrinkle length). Of note, although SCINEXA was validated as a scale which calculates an overall skin aging score from the individual scores of 23 skin aging signs^7^, most studies using SCINEXA (n = 10) reported individual outcome scores, instead of overall skin aging score, while only one study reported overall SCINEXA score^27^. Since skin aging was most frequently assessed as individual outcomes, this review examined the individual outcomes, or skin aging signs, as proxy measures of overall skin aging. For each skin aging sign, we categorised associated risk factors into two types: non-modifiable and modifiable risk factors. Non-modifiable risk factors are associated with intrinsic skin aging and cannot be altered, such as age and gender. In contrast, modifiable risk factors, such as smoking and sun exposure, influence extrinsic skin aging and can be altered through interventions and lifestyle changes.

### Non-modifiable risk factors (intrinsic aging)

#### Age

**Figure 2:**
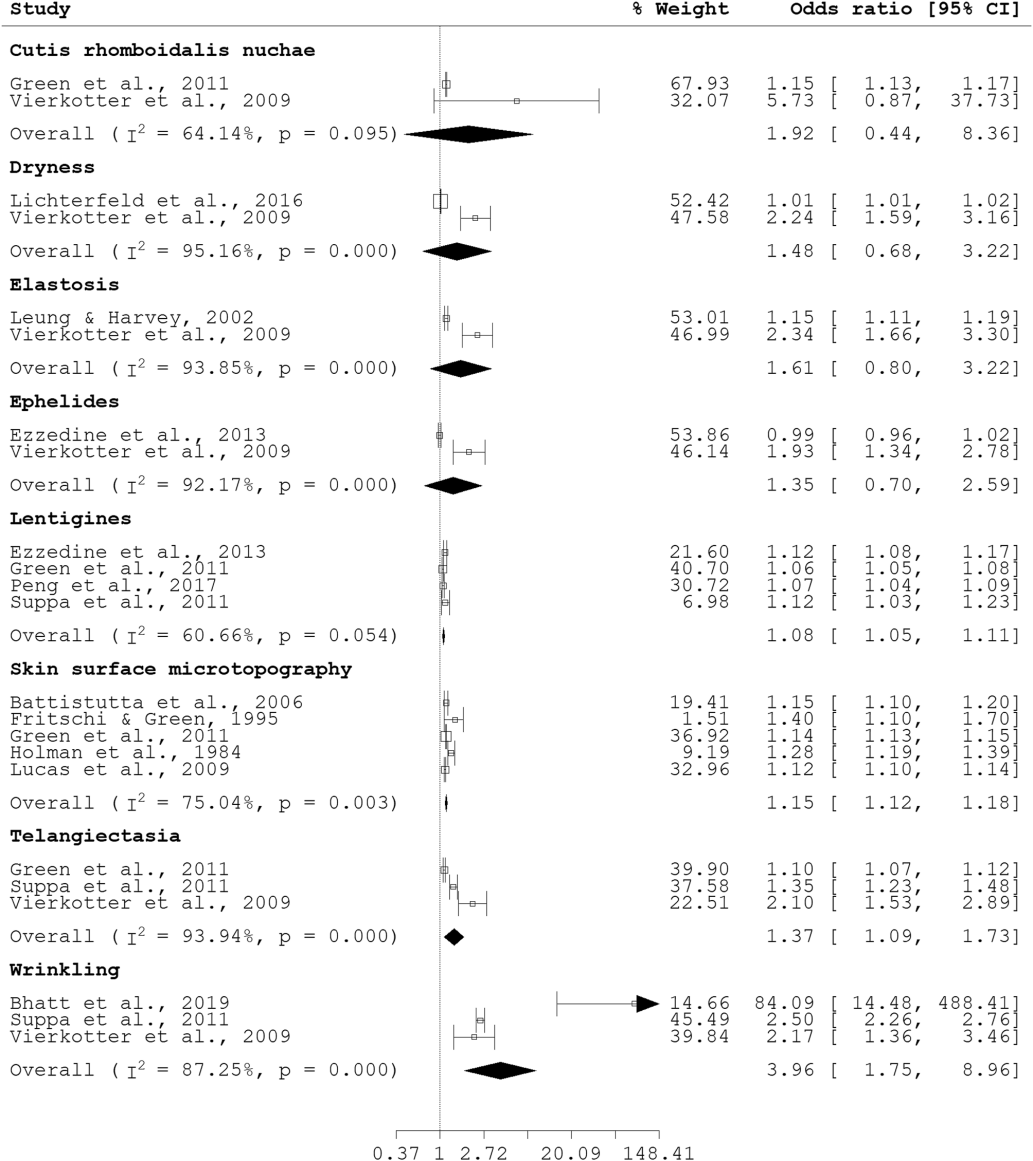
Forest plot for skin aging phenotypes and age as a continuous variable.

Associations with age were obtained for eight skin aging phenotypes: cutis rhomboidalis nuchae, dryness, elastosis, ephelides, facial lentigines, higher SSM score, telangiectasia, and wrinkling (see Fig. 2). Significant associations were found for facial lentigines (pOR 1.08, 95% CI 1.05, 1.11), higher SSM score (pOR 1.15, 95% CI 1.12, 1.18), telangiectasia (pOR 1.37, 95% CI 1.09, 1.73), and wrinkling (pOR 3.96, 95% CI 1.75, 8.96). For the significant pooled associations, significant heterogeneity was observed for higher SSM score (I^2^ = 75.04%, p = 0.003), telangiectasia (I^2^ = 75.04%, p = 0.000), and wrinkling (I^2^ = 87.25%, p = 0.000). Since the progression of age results in the inexorable accumulation of effects from intrinsic factors and damage from extrinsic factors^37^, pooled odds ratio for any given skin aging phenotype with chronological age was likely to be significant. Concordant with expectations, we reported four significant pooled estimates above. However, the pooled estimates for cutis rhomboidalis nuchae, dryness, elastosis, and ephelides respectively with age were non-significant.

#### Gender

**Figure 3:**
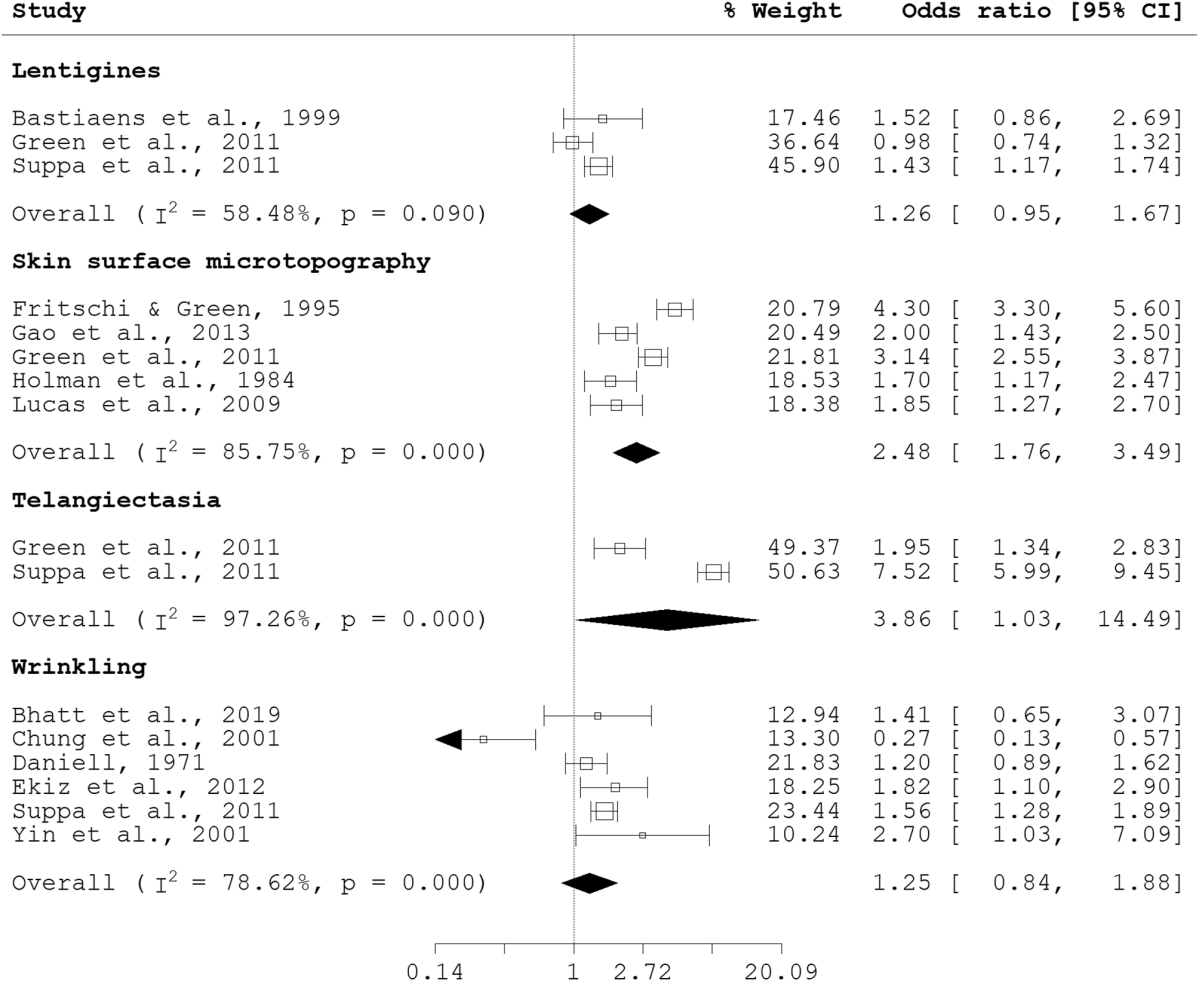
Forest plot for skin aging phenotypes and male gender (female gender as reference).

Pooled associations with male gender were obtained for lentigines (pOR 1.26, 95% CI 0.95, 1.67), higher SSM score (pOR 2.48, 95% CI 1.76, 3.49), telangiectasia (pOR 3.86, 95% CI 1.03, 14.49) and wrinkling (pOR 1.25, 95% CI 0.84, 1.88). Significant heterogeneity was observed for higher SSM score (I^2^ = 85.75%, p = 0.000), telangiectasia (I^2^ = 97.26%, p = 0.000), and wrinkling (I^2^ = 78.62%, p = 0.000); heterogeneity for lentigines was non-significant (see Fig. 3). Although significant associations were reported for female gender with wrinkling (OR 3.69, 95% CI 1.74, 7.84)^38^ and perioral wrinkling^39^, sensitivity analysis by exclusion of each study from the meta-analysis of wrinkling and gender showed that the association with male gender was significantly strengthened when Chung et al.^38^ was excluded (pOR 1.50, 95% CI 1.27, 1.77) (Supplementary Fig. 1).

**Figure 4:**
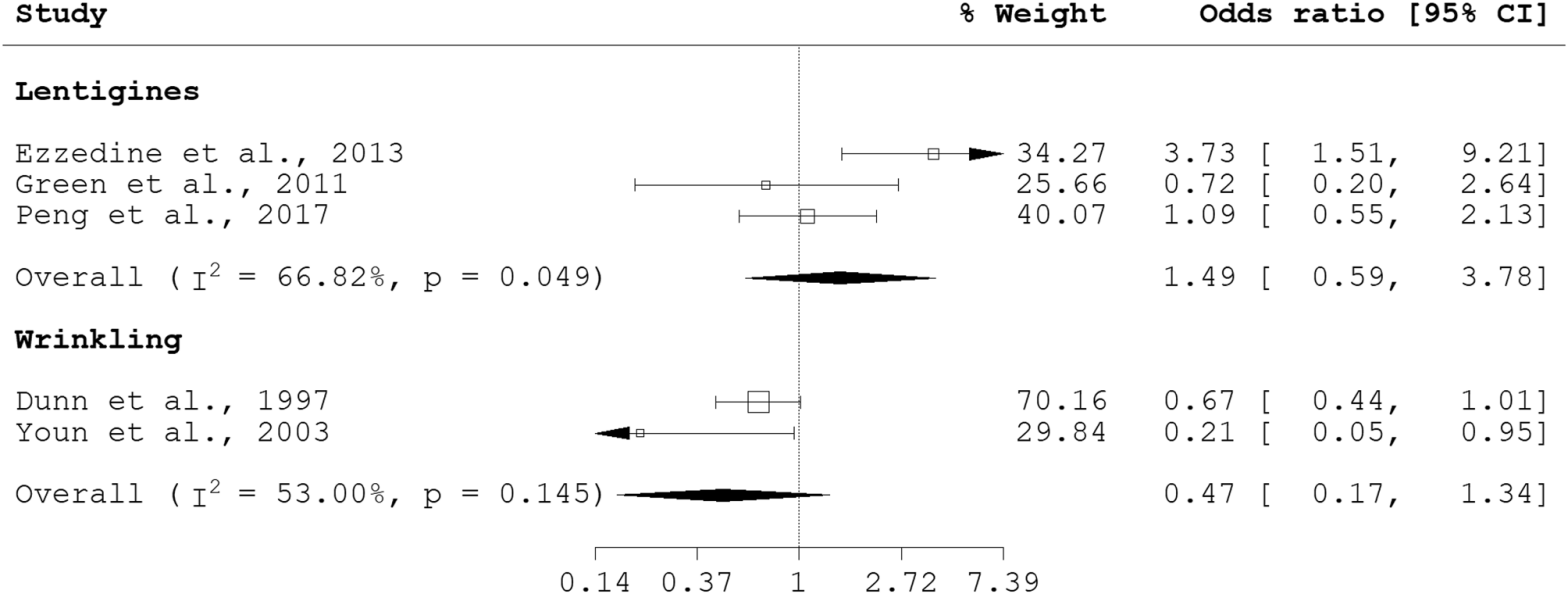
Forest plot for skin aging and HRT use.

Nonetheless, there appears to be a general male predisposition to increase likelihood of skin aging manifestation. While the pathophysiology is unclear, the role of sex hormones has been implicated and reviewed elsewhere^40^. Additionally, menopausal status and hormone replacement therapy (HRT) use, both factors which interact with increased age and female gender, may influence skin aging manifestation. Although no pooled odds ratio was available for menopausal status and skin aging, individual studies have previously reported a significant association for postmenopausal status with photoaging (OR 1.5, 95% CI 1.2, 1.8)^41^, and no significant association with wrinkling (OR 5.00, 95% CI 0.37, 67.66)^25^. Pooled associations with HRT use was obtained for lentigines (pOR 1.49, 95% CI 0.59, 3.78) and wrinkling (pOR 0.47, 95% CI 0.17, 1.34) (see Fig. 4). Notably, although the protective effect of HRT on wrinkling was previously reported^25,35^, the pooled association for wrinkling and HRT use was non-significant.

#### Ethnicity and dyspigmentation

Differences in skin aging progression between ethnicities have been reported across several studies. One study evaluating facial skin aging in African-Americans, Caucasians, Chinese, and Indians found that skin aging manifests differently between each ethnicity, with dyspigmentation being the predominant skin aging trait in Asians^42^. Indeed, a comparison between Chinese and French females showed that increased dyspigmentation occurred more frequently in the Chinese than French, and Chinese females exhibit a marked progression in wrinkling severity at a later age than French females^43^. Likewise, Japanese, when compared to Germans, exhibited more lentigines [arithmetic means ratio (AMR) 6.173, 95% CI 2.959–12.88] and decreased wrinkling severity (Wrinkling under the eyes, AMR 0.813, 95% CI 0.639–0.987; Wrinkling on the upper lip, AMR 0.588, 95% CI 0.274–0.901)^44^. Significant associations for decreased forehead, crow’s feet, glabellar, and perioral wrinkling with Asian ethnicity were also identified^45^ (Supplementary Dataset).

However, a multi-ethnicity study by Vierkötter et al.^46^ concluded that stratification of study subjects by age, anatomical site of skin aging sign, and sub-ethnic group within the Asian category resulted in findings that contradicted the view that skin aging manifestation was specific to ethnicity. In a subject population aged above 30, dyspigmentation was found to be more prevalent in Chinese and Japanese than in Germans only if measured on the cheeks and in subjects up to 60 years old, while wrinkling in Japanese above 60 years old and Chinese of any age were not significantly more severe than that in Germans (Supplementary Dataset). Here, the importance of age in skin aging was underscored, and supported an earlier finding that Chinese have exhibit lower wrinkling severity below 40 years old but show accelerated skin aging from 40 to 50 years old^43^. Indeed, the unequal distribution of subjects within each age range in Vierkötter et al.^46^ likely affected the study’s findings. Moreover, the confounding effect of sub-ethnicity on skin aging phenotype cannot be discounted; one study reported a non-significant difference in wrinkling between Mongolians, an Asian ethnic group, and Caucasians (OR 1.014, 95% CI 0.468–2.196)^34^. Nonetheless, ethnic differences in skin aging have been found to be independent of educational level, sun exposure and smoking^46^.

Skin colour has been correlated with ethnicity, with Europeans having the lightest skin, Africans exhibiting the darkest skin, and Chinese and Indian showing lightness values between the two^47^. However, this review identified no clear association between skin colour and skin aging. The categorisation of skin colour and the corresponding associations with skin aging differed between studies (Supplementary Dataset). Moreover, interaction of sun exposure and skin phototype as determinants of skin colour further complicates the categorisation of skin colour and confounds associations with skin aging phenotypes, particularly in studies that did not explicitly differentiate constitutive and facultative pigmentation. Additional pigmentation-related factors identified by this review include hair and eye colour, for which no clear association could be found by this review owing to inter-study differences in categorisation of exposure and significance of associations.

### Modifiable factors (extrinsic aging)

#### Air pollution

No pooled odds ratio could be obtained for skin aging and air pollution. Each individual study included in this review investigated distinct pollutants in both Asian and Caucasian populations, covering a broad range of air pollutant types overall (Supplementary Table 4). Nonetheless, it appeared that dyspigmentation (or lentigines) and wrinkling on the face were significantly associated with various types of air pollution. Significant associations for increased lentigines were found with Air Quality Index (AQI)^48,49^, contact with fossil fuels^50^, nitrogen dioxide^51^, particulate matter of diameter 2.5 microns or less (PM2.5)^50,52,53^, particulate matter of diameter 10 microns or less (PM10)^54^, second hand smoke exposure^50^, soot^54^, and traffic-associated particles^54^. For increased wrinkling, significant associations were found with AQI^48,49^, cooking with solid fuels leading to indoor pollution^55^, ozone^56^, PM2.5^53^, PM10^54^, soot^54^, and traffic-associated particles^54^. Other significant associations included sagging with AQI^49^, cooking with solid fuels^55^, and PM2.5^53^; and greater perceived age than chronological age with AQI^49^. Of note, non-significant associations were reported by one study for both dyspigmentation and wrinkling with carbon monoxide, ozone, PM2.5, PM10, and sulfur dioxide^57^; these results were attributed to imprecise quantification of air pollutants. Additionally, interaction between pollutants and UV radiation has been reported: nitrogen dioxide, ozone, and UV radiation^56^; particulate matter and UV radiation^52^. Elaborations on pollutant interactions and mechanistic evidence for skin aging and air pollution (e.g., dyspigmentation and particulate matter; wrinkling and ozone) have been reviewed elsewhere^9,10,58–60^.

#### Nutrition

This review found six studies examining the association of skin aging signs with nutritional intake or diet^57,61–65^. The skin aging phenotypes and nutritional exposures investigated varied widely between studies (Supplementary Table 5). A diverse range of food groups and micronutrients were assessed, resulting in different associations being obtained. Only one study used an index to quantify the general dietary intake^63^. Despite the heterogeneity between studies, a healthier dietary intake appears to be associated with less severe skin aging appearance: fatty acids were significantly associated with lower likelihood of dryness, photoaging, and lower SSM score^61,62,65^; increased vegetable consumption was significantly associated with decreased wrinkling and lower SSM score^64,65^; the Dutch Healthy Diet Index (DHDI) was associated with decrease wrinkling^63^. The association of alcohol intake was also examined by multiple studies^21,23,35,36,57,65–70^. As with skin aging and diet associations, no pooled association for skin aging and alcohol consumption could be obtained due to the between-study variation in the quantification of alcohol consumption. Nonetheless, excepting one study which identified a significant association for wrinkling and more than 40 g of alcohol consumed per day (RR 1.35, 95% CI 1.12, 2.97)^35^, all other studies reported non-significant associations for skin aging signs and alcohol consumption.

#### Smoking

**Figure 5:**
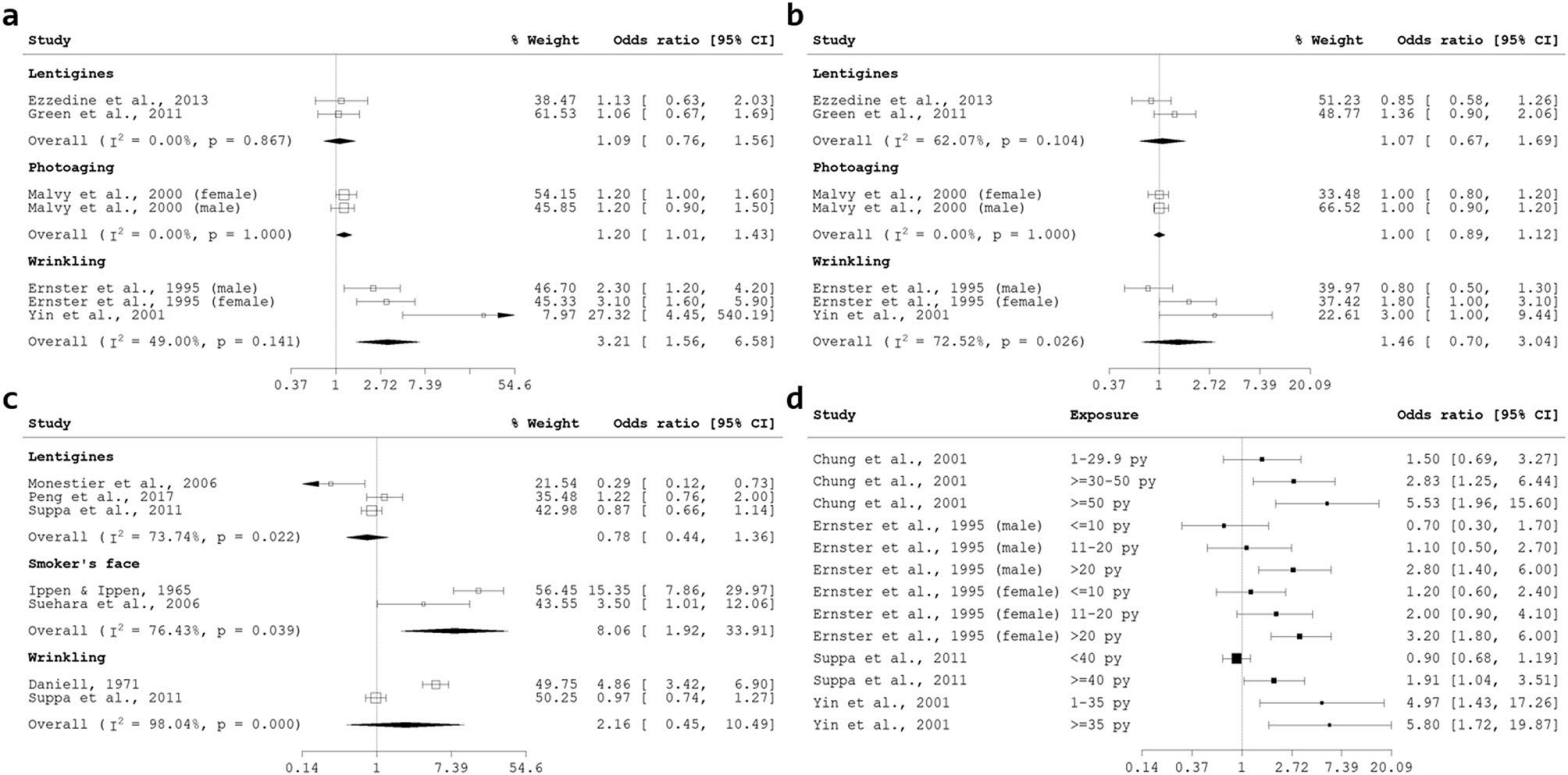
(**a**) Forest plot for skin aging and current smoking (non-smoking as reference). (**b**) Forest plot for skin aging and former smoking (non-smoking as reference). (**c**) Forest plot for skin aging and being a smoker (ever smoking or smoker = yes), with non-smoker as reference. (**d**) Forest plot illustrating dose response relationship for smoking (in pack-years) and wrinkling.

Smoking was categorised as smoking status or smoking exposure. Studies examining smoking status compared ever smokers to never smokers, or between current, former and non-smokers. In contrast, smoking exposure was quantitative, and measured in units of pack-years, total number of years of smoking, cigarettes per day, or cigarettes in a lifetime. For current smoking, the association with lentigines was non-significant (pOR 1.09, 95% CI 0.76, 1.56) with non-significant heterogeneity (I^2^ = 0.00%, p = 0.867). One study reported association for current smoking with photoaging, stratified for gender^41^; pooling the ORs resulted in a significant association (pOR 1.20, 95% CI 1.01, 1.43). Finally, a significant association found for wrinkling and current smoking (pOR 3.21, 95% CI 1.56, 6.58), with non-significant heterogeneity (I^2^ = 49.00%, p = 0.141) (see Fig. 5a). Conversely, no significant association with former smoking was found for either lentigines (pOR 1.07, 95% CI 0.67, 1.69), photoaging (pOR 1.00, 95% CI 0.89, 1.12) or wrinkling (pOR 1.46, 95% CI 0.70, 3.04) (see Fig. 5b). Pooling the associations for skin aging with being a smoker, which referred to subjects who indicated they had ever smoked or yes to being a smoker, resulted in a significant association for smoker’s face only (pOR 8.06, 95% CI 1.92, 33.91) with significant heterogeneity (I^2^ = 76.43%, p = 0.039). Pooled odds ratios for lentigines and wrinkling with being a smoker were both non-significant (see Fig. 5c). Nonetheless, there was a dose–response relationship between pack-years of smoking and wrinkling, across four studies, where an increase in number of pack-years of smoking resulted in a stronger association with wrinkling manifestation (Fig. 5d).

#### UV exposure

**Figure 6:**
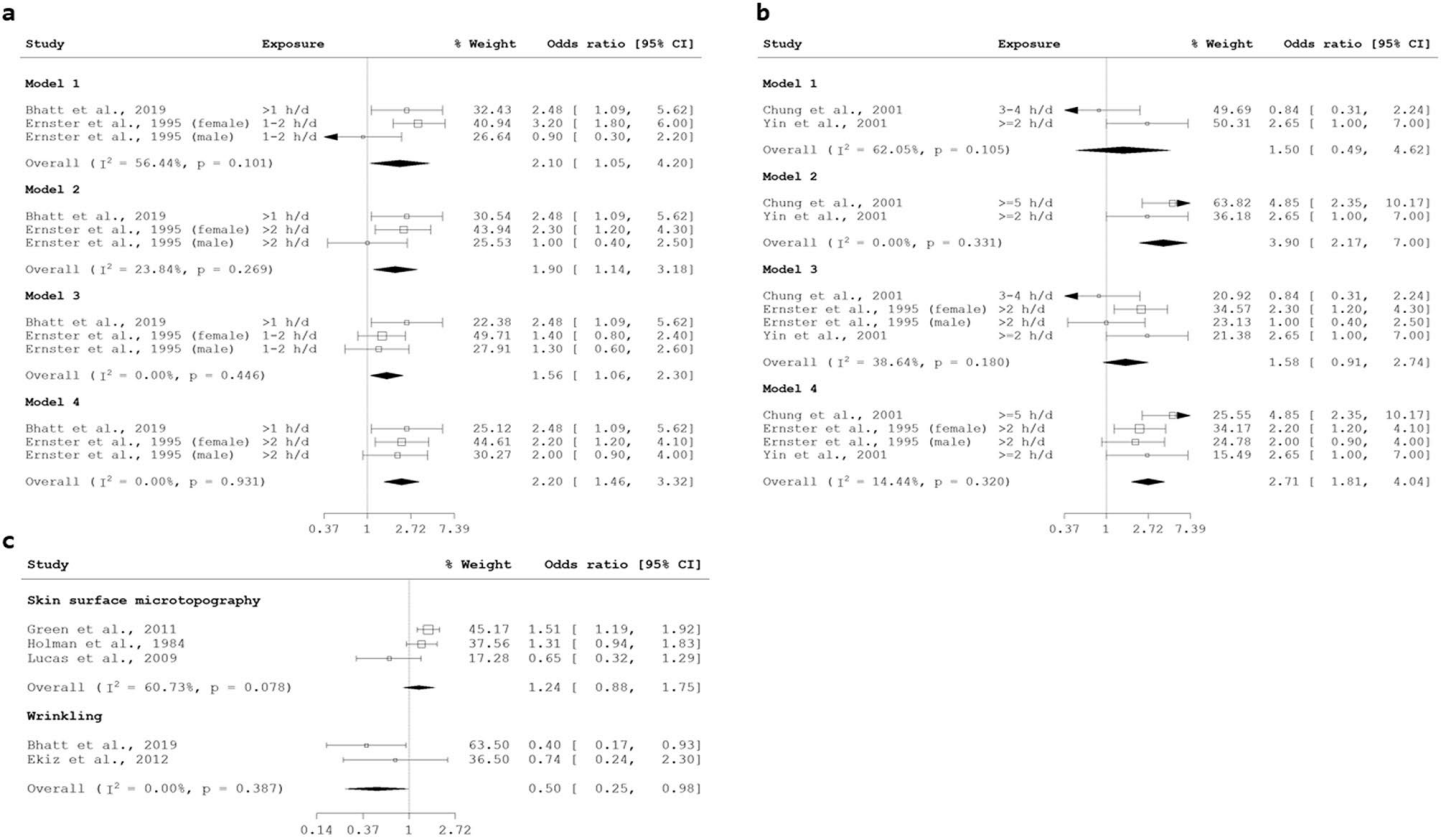
(**a**) Forest plots for more than 1 h/day of sun exposure and skin aging. The comparison exposure for all odds ratio is 1 h/day of sun exposure. Model 1 and 2: Ernster et al.^36^ adjusted for age, BMI, and smoking exposure. Model 3 and 4: Ernster et al.^36^ adjusted for age, BMI, and smoking status. (**b**) Forest plots for more than 2 h/day of sun exposure and wrinkling (compared with less than 2 h/day of sun exposure). Model 1 and 2: Ernster et al.^36^ excluded from models. Model 3 and 4: included Ernster et al., 1995. (**c**) Forest plots for sunscreen use and skin aging.

The contribution of solar UV exposure to skin aging, resulting in a phenotype termed as photoaging has been established^5,6,20^. In the literature, sun exposure was quantified using various units (e.g., cumulative lifetime hours, hours per day) and stratified variously by types of sun exposure (e.g., recreational sun exposure, occupational sun exposure). Between studies using identical units, exposure intensity was categorised differently, thus multiple meta-analyses were conducted. When adjusted for smoking exposure, there was significant association between more than 1 h per day of sun exposure and wrinkling (pOR 1.90, 95% CI 1.14, 3.18) with non-significant heterogeneity (I^2^ = 23.84%, p = 0.269) (Fig. 6a, Model 2). For wrinkling due to more than 2 h per day versus less than 2 h per day, due to various categories reported between studies, only meta-analyses considering a sample with a preponderance of higher sun exposure yielded significant pORs (Fig. 6b). Nonetheless, a dose–response relationship between sun exposure and wrinkling could be discerned. Indeed, for studies that did not report odds ratios, higher degrees of sun exposure were significantly associated with increased wrinkling (Supplementary Table 6). Although pORs for other skin aging signs were unavailable, significant associations with higher grades of sun exposure were observed for several notable phenotypes across multiple studies, including lentigines (or dyspigmentation)^18,71–77^, perceived age^29,78–80^, sagging^74–77^. Interestingly, photoaging, often attributed to sun exposure, was not always significantly associated with sun exposure (Supplementary Table 6).

A related protective factor, sunscreen use, was significantly associated with wrinkling (pOR 0.50, 95% CI 0.25, 0.98) with non-significant heterogeneity (I^2^ = 0.00%, p = 0.387). however, studies that did not report odds ratios found non-significant associations for wrinkling with sunscreen use^39,68,81,82^; another which stratified sunscreen use by reasons for use reporting findings of inconsistent significance^69^. Likewise, the association of sunscreen use with SSM score was non-significant (Fig. 6c). Nonetheless, significant associations were reported for sunscreen use with lower perceived age^68^ and reduced degree of photoaging^30^. Additional factors, such as a dose–response effect of sunscreen use on skin aging, or interaction between sunscreen use and sun exposure may confound the association between sunscreen use and skin aging.

#### Others

Several articles not included in this review have examined exposures worth mentioning, such as the impact of stress on skin aging. Financial stress has been found to result in older perceived age^83^, while the mechanistic and phenotypic effects of stress on the skin have been reviewed, a possible link to older looks has been suggested^84^. Previous studies also found that good sleep was associated with lower SCINEXA intrinsic aging score^27^ (Supplementary Dataset), while sleep deprivation led to a less healthy and less attractive appearance^85^. Another study also found that sleep deprivation led to a perceived increase in facial signs, including hanging eyelids and wrinkling^86^.

## Limitations and conclusion

This review was limited to visible measures of skin aging outcomes, entailing the exclusion of instrumental measurement and biopsy staining methods, which could provide a more accurate measure of phenotype. Dryness has been quantified by skin conductance measured using Skicon-200^87^, while biopsies could permit better quantitative analyses of skin aging via the staining of elastotic tissue^88^, or histologic study^2^. Although studies have taken steps to establish reliability and reproducibility of their various scales by repeating the grading process and assessing the inter-rater reliability, the visual assessment method is inherently subjective and not impervious to cultural perceptions of aged looks. Moreover, since skin aging comprised multiple phenotypes and risk factor exposures were categorised differently between studies, the number of associations obtained by this review for a given skin aging sign and corresponding risk factor were relatively low.

There was a preponderance of cosmetic industry funding and frequent focus on the visuals of skin aging in studies presently reviewed. Although there was no evidence that skin aging phenotypes examined herewith were responsible for functional impairment of the skin nor increased mortality per se, they are nonetheless indicators of histological changes that may contribute to functional impairment of the skin^89,90^. For example, aging of the skin is accompanied by degradation of collagen and elastic fibres in the dermis, thinning of the epidermis, impaired fibroblast function, and other changes reviewed elsewhere^91–94^. These changes have been shown to impair cutaneous integrity, wound healing, and sensory and immune function^94–96^. Moreover, there was an overlap in risk factors for skin aging and skin cancers, with the notable example of UV exposure, thus granting a plausible relevance of skin aging phenotypes as indicators of exposure to risk factors for skin cancer^97–101^.

In summary, we have conducted a systematic review and meta-analysis of skin aging phenotypes and their associated risk factors. Through a reasonably comprehensive literature search, this review has collated a record of skin aging signs and their associated risk factors. Considering the variety of skin aging definitions in the literature, this review has proposed standard definition of skin aging. Of the identified skin aging signs, reports on dyspigmentation, sagging, telangiectasia, and wrinkling were predominant in the literature—an observation we attributed to the visibility and noticeability of said phenotypes. Notably, the most important skin aging phenotype was wrinkling, which was frequently used as an indicator of skin aging and a constituent of both validated and non-validated scales in the literature. Of the intrinsic risk factors for skin aging, the primary influence was age, which was highly associated with wrinkling. Conspicuous extrinsic aging factors were smoking and sun exposure, both of which were significantly associated with multiple skin aging signs and exhibited dose-responses relationships with wrinkling.

Finally, this article will serve as a rough directory to the relevant original research publications at bare minimum, while providing readers with an overview of signs linked to skin aging and their associated risk factors. Besides examining risk factors with established significant associations with skin aging, we have also put forward some associations that deserve closer scrutiny. The risk factors identified herewith will guide future research, such as genetic association studies, wherein the interplay between environmental influences and genetics are elucidated.

## Supplementary Information

Supplementary Information 1.

Supplementary Information 2.
