# Supplementary material for: Defining skin aging and its risk factors: a systematic review and meta-analysis
Source: Sci Rep. 2021 Nov 11;11:22075. doi: 10.1038/s41598-021-01573-z (PMC8586245; doi:10.1038/s41598-021-01573-z)
Supplement: Supplementary file 2 — Supplementary Information 2. [file 41598_2021_1573_MOESM2_ESM.docx]

Supplementary Table 1. PRISMA checklist for systematic review and meta-analysis. (From Moher D, Liberati A, Tetzlaff J, Altman DG, The PRISMA Group (2009). Preferred Reporting Items for Systematic Reviews and Meta-Analyses: The PRISMA Statement. PLoS Med 6(7): e1000097. doi:10.1371/journal.pmed1000097)

| **SECTION/TOPIC** | **#** | **CHECKLIST ITEM** | **REPORTED ON PAGE #** |
| --- | --- | --- | --- |
| **TITLE** | | | |
| Title | 1 | Identify the report as a systematic review, meta-analysis, or both. | Title |
| **ABSTRACT** | | | |
| Structured summary | 2 | Provide a structured summary including, as applicable: background; objectives; data sources; study eligibility criteria, participants, and interventions; study appraisal and synthesis methods; results; limitations; conclusions and implications of key findings; systematic review registration number. | Abstract |
| **INTRODUCTION** | | | |
| Rationale | 3 | Describe the rationale for the review in the context of what is already known. | Section 3.1 |
| Objectives | 4 | Provide an explicit statement of questions being addressed with reference to participants, interventions, comparisons, outcomes, and study design (PICOS). | Section 3.2 and 3.3 |
| **METHODS** | | | |
| Protocol and registration | 5 | Indicate if a review protocol exists, if and where it can be accessed (e.g., Web address), and, if available, provide registration information including registration number. | NA |
| Eligibility criteria | 6 | Specify study characteristics (e.g., PICOS, length of follow-up) and report characteristics (e.g., years considered, language, publication status) used as criteria for eligibility, giving rationale. | Section 4.1 |
| Information sources | 7 | Describe all information sources (e.g., databases with dates of coverage, contact with study authors to identify additional studies) in the search and date last searched. | Section 4.1 |
| Search | 8 | Present full electronic search strategy for at least one database, including any limits used, such that it could be repeated. | Section 4.1 |
| Study selection | 9 | State the process for selecting studies (i.e., screening, eligibility, included in systematic review, and, if applicable, included in the meta-analysis). | Section 4.2 |
| Data collection process | 10 | Describe method of data extraction from reports (e.g., piloted forms, independently, in duplicate) and any processes for obtaining and confirming data from investigators. | Section 4.3 |
| Data items | 11 | List and define all variables for which data were sought (e.g., PICOS, funding sources) and any assumptions and simplifications made. | Section 4.3 |
| Risk of bias in individual studies | 12 | Describe methods used for assessing risk of bias of individual studies (including specification of whether this was done at the study or outcome level), and how this information is to be used in any data synthesis. | Section 4.2 |
| Summary measures | 13 | State the principal summary measures (e.g., risk ratio, difference in means). | Section 4.3 |
| Synthesis of results | 14 | Describe the methods of handling data and combining results of studies, if done, including measures of consistency (e.g., I^2^) for each meta-analysis. | Section 4.4 |
| Risk of bias across studies | 15 | Specify any assessment of risk of bias that may affect the cumulative evidence (e.g., publication bias, selective reporting within studies). | Section 4.4 |
| Additional analyses | 16 | Describe methods of additional analyses (e.g., sensitivity or subgroup analyses, meta-regression), if done, indicating which were pre-specified. | NA |
| **RESULTS** | | | |
| Study selection | 17 | Give numbers of studies screened, assessed for eligibility, and included in the review, with reasons for exclusions at each stage, ideally with a flow diagram. | Section 5.1 |
| Study characteristics | 18 | For each study, present characteristics for which data were extracted (e.g., study size, PICOS, follow-up period) and provide the citations. | Section 5.2 |
| Risk of bias within studies | 19 | Present data on risk of bias of each study and, if available, any outcome level assessment (see item 12). | Section 5.1 |
| Results of individual studies | 20 | For all outcomes considered (benefits or harms), present, for each study: (a) simple summary data for each intervention group (b) effect estimates and confidence intervals, ideally with a forest plot. | Section 5.5 and 5.6 |
| Synthesis of results | 21 | Present results of each meta-analysis done, including confidence intervals and measures of consistency. | Section 5.5 and 5.6 |
| Risk of bias across studies | 22 | Present results of any assessment of risk of bias across studies (see Item 15). | Section 5.5 and 5.6 |
| Additional analysis | 23 | Give results of additional analyses, if done (e.g., sensitivity or subgroup analyses, meta-regression [see Item 16]). | NA |
| **DISCUSSION** | | | |
| Summary of evidence | 24 | Summarize the main findings including the strength of evidence for each main outcome; consider their relevance to key groups (e.g., healthcare providers, users, and policy makers). | Section 5.5 and 5.6 |
| Limitations | 25 | Discuss limitations at study and outcome level (e.g., risk of bias), and at review-level (e.g., incomplete retrieval of identified research, reporting bias). | Section 6 |
| Conclusions | 26 | Provide a general interpretation of the results in the context of other evidence, and implications for future research. | Section 5 and 6 |
| **FUNDING** | | | |
| Funding | 27 | Describe sources of funding for the systematic review and other support (e.g., supply of data); role of funders for the systematic review. | Section 9 |

Supplementary Table 2. Summary of subject characteristics and outcomes reported in studies included in this systematic review.

| **Study (reference)** | **Country** | **Subject characteristics** | **Relevant outcome(s) (term used in article, if different, indicated in parentheses)** | **Outcome grading** |
| --- | --- | --- | --- | --- |
| Aizen & Gilhar, 2001^1^ | NR | 80 males and females, 59-91 years, race NR | Wrinkles | Graded 1-5 |
| Akiba et al., 1999^2^ | Japan (Akiba, Kagoshima) | 195 males and females, 20-29 and 50-59 years, race NR | Pigmentation (hyper-pigmentation) Wrinkles Skin colour (sun exposure index for darkness) | Graded 1-5, using images captured by FaCIS Recorded count and total length using WIS Derived from L*A*B* measurements |
| Allen et al., 1973^3^ | US | 650 males and females, 20-80 years, Whites and Blacks | Wrinkles | Graded 1-6 (Daniell scale) |
| Asakura et al., 2009^4^ | Japan | 802 males and females, >65 years, Asian | Pigmentation (hyperpigmented macules) Pores Texture Wrinkles | Quantified using software Quantified using software Quantified using software Quantified using software Quantified using software |
| Bastiaens et al., 1999^5^ | Netherlands (Leiden) | 272 males and females, 23-69 years, race NR | Ephelides  Lentigines (solar lentigines) | Counted, then graded 1-6 according to frequency Counted, then graded 1-6 according to frequency |
| Bastiaens et al., 2004^6^ | Netherlands (Leiden) | 961 males and females, 30-80 years, race NR (Leiden skin cancer study cohort) | Ephelides Lentigines (solar lentigines) | Presence or absence Presence or absence |
| Battistutta et al., 2006^7^ | Australia (Nambour) | 195 males and females, 18-79 years, race NR | Skin surface microtopography | Graded 1-6 (Beagley-Gibson scale) |
| Buendía-Eisman et al., 2020^8^ | Spain | 1,474 males and females, 18-60 years, race NR | Photoaging (degree of aging) | Graded 1-4 (Glogau scale) |
| Bhatt et al., 2019^9^ | Nepal (Dharan) | 110 males and females, 30-70 years, Asians and Caucasians | Wrinkles | Calculated using formula (from Castelo-Branco et al., 1998) |
| Castelo-Branco et al., 1998^10^ | Spain (Barcelona) | 730 females, 40-60 years, race NR | Wrinkles | Calculated using formula |
| Chien et al., 2016^11^ | US (Michigan) | 143 males and females, 21-91 years, various races | Wrinkles | Graded 0-8 |
| Chien et al., 2018^12^ | US (Baltimore) | 75 females, 18-96, Blacks | Photoaging | Graded 0-8 |
| Chung et al., 2001^13^ | South Korea | 407 males and females, 30-92 years, Asian | Pigmentation (dyspigmentation) Wrinkles Pigmentation (hyper-pigmented macules) Seborrheic keratosis | Graded 0-5 Graded 0-7 Count  Count |
| Cosgrove et al., 2007^14^ | US | 3,808 females, 40-74 years, various races (NHANES I cohort) | Dryness Thinning (skin atrophy) Wrinkles | Presence or absence Presence or absence Presence or absence |
| Daniell, 1971^15^ | US (California) | 1,104 males and females, 30-70 years, 98% white | Wrinkles | Graded 1-6, Daniell scale |
| Derancourt et al., 2007^16^ | France | 285 males and females, 18-58 years, Caucasian | Lentigines (solar lentigines) | Count |
| Ding et al., 2017^17^ | China (Taizhou) | 874 males and females, 35-89 years, Asian, recruited in 2012 from Taizhou cohort; 1,003 males and females, 56-74 years, Asian, recruited in 2014 | Pigmentation (pigment spots)  Sagging (laxity) Wrinkles (coarse wrinkles) Cutis rhomboidalis nuchae Elastosis (solar elastosis) Favre-racouchot syndrome Skin colour (even pigmentation) Telangiectasia Wrinkles (fine wrinkles) | Counted, then graded 0-3 according to count; graded 0-5 according to severity (SCINEXA) Graded 0-5 Graded 0-5 Presence or absence Presence or absence Presence or absence Presence or absence Presence or absence Presence or absence |
| Dobos et al., 2015^18^ | Germany (Berlin) | 24 females, 30-80 years, race NR | Pigmentation (hyperpigmentation)  Pigmentation (hypopigmentation)  Pigmentation (dyspigmentation) | Graded 0-5 and quantified using digital image analysis Graded 0-5 and quantified using digital image analysis Graded 0-5 and quantified using digital image analysis |
| Dunn et al., 1997^19^ | US | 3,875 females, 40-74 years, Whites and Blacks (NHANES I cohort) | Dryness Thinning (skin atrophy) Wrinkles | Presence or absence Presence or absence Presence or absence |
| Ekiz et al., 2012^20^ | Turkey (Ankara) | 574 males and females, 18-89 years, race NR | Wrinkles | Graded 1-6, Daniell scale |
| Elfakir et al., 2010^21^ | France (Paris) | 530 females, 44-70 years, Whites (SUVIMAX cohort) | Photoaging | Graded 1-6, Lanier scale |
| Engel et al., 1988^22^ | US | 20,295 males and females, 1-74 years, Whites and Blacks (NHANES I cohort) | Photoaging (actinic skin damage) Elastosis (senile elastosis) Ephelides (freckles) Solar keratosis (actinic keratosis) Lentigines (senile lentigines) Pigmentation (localised hypermelanism) Pigmentation (localised hypomelanism) Telangiectasia (fine telangiectasia) | Presence or absence Graded absent, minimal, moderate, or severe Graded absent, minimal, moderate, or severe Graded absent, minimal, moderate, or severe Graded absent, minimal, moderate, or severe Graded absent, minimal, moderate, or severe  Graded absent, minimal, moderate, or severe  Graded absent, minimal, moderate, or severe |
| Ernster et al., 1995^23^ | US (California) | 1,136 males and females, 30-69 years, Whites | Wrinkles | Calculated by assigning 1, 1.5 or 2 to shallow, medium, and deep wrinkles, respectively; then taking length of each wrinkle in cm; multiply both numbers; add the scores for all wrinkles at the locations of interest |
| Eun, 2001^24^ | NR | 407 males and females, 30-92 years, Asian | Wrinkles | Graded 0-7 |
| Ezzedine et al., 2013^25^ | France (Paris) | 523 females, 44-70 years, race NR (SUVIMAX cohort) | Ephelides Lentigines | Presence or absence Graded using a six-grade scale by Morizot et al., 2002. Global severity was estimated by a score built using principal component analysis and linear regression and standardised |
| Flament & Qiu, 2017^26^ | China (Shanghai) | 83 females, age NR, Asian | Pigmentation  Pores Sagging Telangiectasia (vascular disorders) Wrinkles | Graded according to Skin Aging Atlas. Volume 2. Asian Type. (Bazin & Flament, 2010) |
| Flament et al., 2013^27^ | France (Montpellier) | 298 females, 30-78 years, white | Wrinkles sagging pigmentation  Telangiectasia (vascular disorders) | Graded according to Skin Aging Atlas. Volume 1. Caucasian Type. (Bazin & Flament, 2007) |
| Flament et al., 2015^28^ | China (Guangzhou) | 301 female, 20-80 years, Asian | Pigmentation  pores sagging wrinkles | Graded according to skin aging atlas. Volume 2. Asian type. (Bazin & Flament, 2010) |
| Flament et al., 2018^29^ | China (Baoding, Dalian) | 204 females, 25-45 years, Asian | Pigmentation  Pores Sagging Telangiectasia (vascular disorders) Wrinkles | Graded according to Skin Aging Atlas. Volume 2. Asian Type. (Bazin & Flament, 2010) |
| Flament et al., 2019^30^ | China (Baoding, Dalian) | 201 males, 20-60 years, Chinese | Pigmentation  Pores (sebaceous pores) Sagging Telangiectasia (vascular disorders) Wrinkles | Graded according to Skin Aging Atlas. Volume 2. Asian Type. (Bazin & Flament, 2010) |
| Flament et al., 2019^31^ | Japan (Tokyo) | 1,011 females, 18-83 years, Asian | Pigmentation  Sagging Telangiectasia (vascular disorders) Wrinkles | Graded according to Skin Aging Atlas. Volume 2. Asian Type. (Bazin & Flament, 2010) |
| Flament et al., 2019^32^ | China (Hong Kong, Shanghai) | 261 males, 18-75 years, Asian | Pigmentation  Pores Sagging Telangiectasia (vascular disorders) Wrinkles | Graded according to Skin Aging Atlas. Volume 2. Asian Type. (Bazin & Flament, 2010) |
| Fritschi & Green, 1995^33^ | Australia; Scotland | 1307 males and females, 13-15 years, race NR | Skin surface microtopography | Graded none, mild moderate, or severe |
| Fuks et al., 2019^34^ | Germany (Berlin, Ruhr) | 2013 males and females, 20-84 years, race NR (SALIA and BASE-II cohort) | Wrinkles (coarse wrinkles) Pigmentation (pigment spots) | Graded according to SCINEXA Graded according to SCINEXA |
| Gao et al., 2013^35^ | China (Hainan, Liaoning) | 691 males and females, 10-86 years, Asian | Skin surface microtopography | Graded 1-6, Beagley-Gibson scale |
| Garbe et al., 1994^36^ | Germany | 1,011 males and females, age and race NR | Lentigines (actinic lentigines) | Graded none, few, or many |
| Gill et al., 2000^37^ | Australia (Melbourne) | 170 males and females, 15-30 years, various races | Seborrheic keratosis | Count |
| Goodman et al., 2018^38^ | Australia; Canada; UK; US | 1,472 females, 18-75 years, Caucasians and Asians | Wrinkles (lines) Wrinkles (nasolabial fold) Others (midface volume loss) Others (oral commissures) Others (tear troughs) | Graded 0-3 Graded 0-4 Graded 0-5 Graded 0-3 Graded 0-4 |
| Green et al., 1988^39^ | Australia (Nambour) | 834 males and females, 20-69 years, race NR | Seborrheic keratosis | Count |
| Green et al., 2011^40^ | Australia (Nambour) | 1,400 males and females, 20-54 years, race NR | Skin surface microtopography Lentigines (solar lentigines) Telangiectasia Cutis rhomboidalis nuchae Favre-racouchot syndrome | Graded 1-6, Beagley-Gibson scale Count Graded as mild, moderate, or severe Graded as mild to moderate, or severe Graded as mild to moderate, or severe |
| Green, 1991^41^ | Australia (Nambour) | 807 males and females, 20-55 years, race NR | Skin surface microtopography | Graded 1-6, Beagley-Gibson scale |
| Gunn et al., 2015^42^ | Netherlands (Leiden)  UK | 670 males and females, age and race NR (Leiden Longevity Study cohort)  162 females, 45-75 years, Whites | Perceived age (perceived facial age) Wrinkles  Perceived age (perceived facial age) | Estimated in years Graded 1-9  Estimated in years |
| Guyuron et al., 2009^43^ | US | 196 (98 twin pairs) males and females, 18-76 years, race NR | Perceived age (perceived age difference) | Estimated in years |
| Hamer et al., 2017^44^ | Netherlands (Rotterdam) | 3,831 males and females, >45 years, race NR (Rotterdam study cohort) | Wrinkles | Quantified using digital image analysis |
| Helfrich et al., 2007^45^ | US | 82 males and females, 22-91 years, various races | Wrinkles (fine wrinkles) | Graded 0-8 |
| Hillebrand et al., 2001^46^ | Japan (Akita, Kagoshima) | 602 females, 5-65 years, Asian | Pigmentation Wrinkles | Quantified using FaCIS Quantified using WIS |
| Holman et al., 1984^47^ | Australia (Busselton) | 1,216 males and females, 16-86 years, race NR | Skin surface microtopography | Graded 1-6, Beagley-Gibson scale |
| Hüls et al., 2016^48^ | China (Taizhou) | 1,072 males and females, 27-90 years, Asian; 806 females, 66-79 years, Caucasian (SALIA cohort) | Lentigines | Counted, then graded 0, 5, 30, or 75 according to frequency category (SCINEXA) |
| Hüls et al., 2018^49^ | Germany (Ruhr) | 799 females, 69-79 years, Caucasian | Lentigines | Counted, then graded 0-3 based on frequency category (SCINEXA) |
| Ichibori et al., 2014^50^ | Japan (Osaka) | 134 (67 twin pairs) males and females, 40-87 years, Asian | Permanent erythema (erythema) Pigmentation (spots) Pores Texture Wrinkles | Quantified using VISIA Quantified using VISIA Quantified using VISIA Quantified using VISIA Quantified using VISIA |
| Ippen & Ippen, 1965^51^ | NR | 224 females, 35-84 years, race NR | Smoker's face (smoker’s skin) | Presence or absence |
| Jacobs et al., 2014^52^ | Netherlands (Rotterdam) | 5,578 males and females, over 45 years, race NR (Rotterdam Study cohort) | Sagging | Graded as normal, mild, moderate, or severe |
| Kadunce et al., 1991^53^ | US (Utah) | 132 males and females, 35-59 years, Whites | Wrinkles | Graded 1-5, modified from Daniell, 1971 |
| Kennedy et al., 2003^54^ | Netherlands (Leiden) | 956 males and females, 30-80 years, race NR (Leiden Skin Cancer Study cohort) | Elastosis Telangiectasia | Graded as light, moderate, or severe Graded as light, moderate, or severe |
| Kennedy et al., 2003^55^ | Netherlands (Leiden) | 966 males and females, 30-80 years, race NR (Leiden Skin Cancer Study cohort) | Solar keratosis (actinic keratosis) | Count |
| Keough et al., 1997^56^ | US (Texas) | 56 males and females, 37-90 years, race NR | Favre-racouchot syndrome | Presence or absence |
| Kim et al., 2014^57^ | China (Beijing, Shanghai, Wuhan, Guangzhou) | 441 females, 20-35 years, Asian | Wrinkles | Quantified using Visioline |
| Kimlin & Guo, 2012^58^ | Australia (Brisbane) | 180 males and females, 18-83 years, race NR | Pigmentation (hyperpigmentation) Wrinkles | Quantified using VISIA Quantified using VISIA |
| Knuutinen et al., 2002^59^ | Finland | 98 males, 34-71 years, race NR | Thinning (thickness) | Quantified using ultrasound device |
| Koh et al., 2002^60^ | South Korea | 350, gender NR, 20-69 years, race NR | Wrinkles | Graded 1-5, modified from Daniell, 1971 |
| Kwon et al., 2003^61^ | South Korea | 303 males, 40-70 years, Asian | Seborrheic keratosis | Count |
| Latreille et al., 2013^62^ | France (Paris) | 2,919 males and females, 45-60 years, race NR (SUVIMAX cohort) | Photoaging (skin photodamage) | Graded 1-6, Larnier scale |
| Leung & Harvey, 2002^63^ | UK (Cardiff) | 560 males and females, 60-97 years, race NR | Elastosis | Graded 0-10, adapted from Daniell scale |
| Li et al., 2015^64^ | China (Pingding, Taizhou) | 1,129 females, 30-90 years, Asian | Pigmentation (pigment spots)  Sagging (laxity) Wrinkles (coarse wrinkles) Cutis rhomboidalis nuchae Elastosis (solar elastosis) Favre-racouchot syndrome Skin colour (even pigmentation) Telangiectasia Wrinkles (fine wrinkles) | Counted, then graded 0-3 according to frequency category (modified SCINEXA) Graded 0-5 (SCINEXA) Graded 0-5 (SCINEXA) Presence or absence Presence or absence Presence or absence Presence or absence Presence or absence Presence or absence |
| Lichterfeld et al., 2016^65^ | Germany | 1,524 males and females, elderly subjects, age and race NR | Dryness | Graded 0-4 (overall dry skin score) |
| Lucas et al., 2009^66^ | Australia | 534 males and females, 18-61 years, race NR (Ausimmune study cohort) | Skin surface microtopography | Graded 1-6 (Beagley-Gibson scale) |
| Malvy et al., 2000^67^ | France | 6,630 males and females, 45-60 years, race NR (SUVIMAX cohort) | Photoaging (skin photodamage) | Graded 1-6 (Larnier scale) |
| Martires et al., 2009^68^ | US (Ohio) | 130 (65 twin pairs) males and females, 18-77 years, race NR, twins | Photoaging | Graded 0-9, (scale from Griffiths et al., 1992) |
| Mayes et al., 2010^69^ | China (Shanghai) | 220 females, 25-70 years, Chinese | Perceived age (perceived facial age) | Estimated in years |
| Mekic et al., 2019^70^ | Netherlands (Rotterdam) | 2,753 males and females, median age 67.3, race NR (Rotterdam study cohort) | Wrinkles | Quantified digitally using Matlab |
| Mekic et al., 2019^71^ | Netherlands (Rotterdam) | 5,547 males and females, 51-101 years, race NR (Rotterdam Study cohort) | Dryness (dry skin) | Presence or absence |
| Mekić et al., 2020^72^ | Netherlands (Rotterdam) | 2,842 males and females, >45 years, race NR (Rotterdam Study cohort) | Telangiectasia | Quantified using image analysis system |
| Mizuno et al., 2016^73^ | Japan (Wakayama) | 14 males and females, 62-91 years, Asian | Pigmentation (spots) Wrinkling | Quantified using image analysis system Quantified using image analysis system |
| Model, 1985^74^ | NR | 116 males and females, 35-69 years, Whites and 1 Asian | Smoker's face | Presence or absence |
| Monestier et al., 2006^75^ | France (Marseille) | 236 males and females, 60-79 years, Caucasian | Lentigines (senile lentigo) | Count |
| Muizzuddin et al., 1997^76^ | US (New York, New Jersey, Pennsylvania) | 100, no other data reported | Wrinkles | Quantified using image analysis system |
| Nagata et al., 2010^77^ | Japan (Gifu) | 716 females, 20-74 years, Asian | Wrinkles | Graded 1-6 (Daniell scale) |
| Nouveau-Richard et al., 2005^78^ | China (Suzhou); France (Besancon) | 320 females, 20-60 years, race NR | Pigmentation (pigmented spots) Wrinkles | Graded none, few, or many Graded none, few, or many |
| O'Hare et al., 1999^79^ | US (Winston-Salem) | 200 males and females, 35-75 years, Whites | Wrinkles | Graded 0-5 (modified Daniell scale) |
| Oyetakin‐White et al., 2015^80^ | US (Ohio) | 60 females, 30-49 years, race NR | Overall skin aging (skin aging) | Graded according to SCINEXA |
| Okada et al., 2013^81^ | US (Ohio) | 158 (79 twin pairs) males and females, 18-78 years, race NR, twins | Wrinkles | Graded 0-5 (Lemperle assessment scale) |
| Park et al., 2019^82^ | South Korea (Seoul) | 188 males and females, 21-66 years, race NR | Pigmentation Wrinkles | Quantified via image analysis (smartphone app which graded 0-100) |
| Peng et al., 2017^83^ | China (Beijing) | 400 females, 40-90 years, Asian | Lentigines (senile lentigo) Seborrheic keratosis | Counted, then graded 0, 5, 30, or 75 based on frequency (SCINEXA from Li et al., 2015) |
| Perner et al., 2011^84^ | Germany (Düsseldorf); Japan (Nagoya) | 87 females, age NR, Asian and Caucasian | Lentigines Wrinkles | Graded according to SCINEXA Graded according to SCINEXA |
| Purba et al., 2001^85^ | Australia; China; Greece; Japan; Sweden | 453 males and females, >70 years, race NR | Skin surface microtopography | Graded 1-6 (Beagley-Gibson scale) |
| Qiu et al., 2011^86^ | China (Shanghai) | 354 females, 18-80 years, Chinese | Pigmentation (pigmentation spot) Sagging Wrinkles | Quantified using mexameter NR graded 0-6 |
| Raduan et al., 2008^87^ | Brazil (Rio de Janeiro) | 301 males and females, 25-86 years, race NR | Wrinkles | Graded 1-6, Daniell scale |
| Raitio et al., 2004^88^ | Finland (northern region) | 89 males, 15-56 years, race NR | Wrinkles | Graded 1-6 ( Daniell scale); also quantified using computer analysis |
| Rexbye et al., 2006^89^ | Denmark | 1,826 males and females, 63-90 years, race NR (LSADT 2001 cohort) | Perceived age | Estimated in years |
| Schäfer et al., 2006^90^ | Germany (Augsburg) | 2,823 males and females, 25-74 years, race NR (KORA-survey 2000 cohort) | Solar keratosis Cutis rhomboidalis nuchae Elastosis Favre-racouchot syndrome Ephelides Lentigines (lentigines solaris) Lentigines (lentigines senilis) | Presence or absence Graded absent, mild, moderate, or severe Graded absent, mild, moderate, or severe Graded absent, mild, moderate, or severe Count Count Count |
| Seddon et al., 1992^91^ | US (Massachusetts) | 115 males and females, 18-77 years, race NR | Skin surface microtopography | Graded 1-6 (Beagley-Gibson scale) |
| Silva et al., 2009^92^ | UK (Hertfordshire, Yorkshire) | 754 females, 18-46 years, white | Skin surface microtopography | Graded 1-6 (Beagley-Gibson scale) |
| Singer et al., 1994^93^ | US (Michigan) | 120 males and females, 43-81 years, race NR | Photoaging (photodamage) | Graded 0-8 (scale from Griffiths et al., 1992) |
| Song et al., 2015^94^ | South Korea (Seoul, Gyeonggi-do) | 100 males, 20-59 years, Asian | Wrinkles | Quantified using PRIMOS lite |
| Suehara et al., 2006^95^ | Brazil (Sao Paulo) | 77 males and females, 41-60 years, race NR | Smoker's face | Graded 1-7 |
| Suppa et al., 2011^96^ | UK (Yorkshire, Northern Region south of the River Tyne') | 1,342 males and females, 18-76 years, race NR | Pigmentation Telangiectasia (vascularity) Wrinkles | Graded 0-5 Graded 0-4 Graded 0-7 |
| Takahashi et al., 2017^97^ | Japan (Tokyo) | 169 females, 6-62 years, Asian | Pigmentation | Quantified using VISIA |
| Tsukahara et al., 2007^98^ | Japan (Tokyo); China (Shanghai); Thailand (Bangkok) | 277 females, 10-70 years, Asians | Sagging Wrinkles | Graded 0-5 Graded 0-5 |
| Tsukahara et al., 2013^99^ | Japan (Tochigi) | 173 males and females, 21-75 years, Asian | Wrinkles | Graded 1-5 |
| Tsukahara et al., 2013^100^ | Japan (Tokyo) | 32 males and females, 35-47 years, Asian | Wrinkles | Graded 1-5 |
| Vierkötter et al., 2009^101^ | Germany (North Rhine Westphalia) | 74 males and females, 19-72 years, race NR | Comedones Dryness Elastosis Ephelides (sunburn freckles) Lentigines (lentigines solaris) Others (change of skin phototype) Others (pigment change) Others (pseudoscars) Others (reduced fat tissue) Others (yellowness) Permanent erythema Sagging (lax appearances) Seborrheic keratosis (benign skin tumour) Telangiectasia Wrinkles (coarse wrinkles) Wrinkles (fine wrinkles) Cancer (basal cell carcinoma) Cancer (malignant melanoma) Cancer (squamous cell carcinoma) Cutis rhomboidalis nuchae Favre-racouchot syndrome Solar keratosis (actinic precancerosis) Pigmentation (uneven pigmentation) | Graded 0-3 Graded 0-3 Graded 0-3 Graded 0-3 Graded 0-3 Graded 0-3 Graded 0-3 Graded 0-3 Graded 0-3 Graded 0-3 Graded 0-3 Graded 0-3 Graded 0-3  Graded 0-3 Graded 0-3 Graded 0-3 Presence or absence Presence or absence Presence or absence Presence or absence Presence or absence Presence or absence  Presence or absence |
| Vierkötter et al., 2010^102^ | Germany (Ruhr, Borken) | 400 females, 68-79 years, race NR (SALIA study cohort) | Pigmentation (pigment spots) Seborrheic keratosis Elastosis (solar elastosis) Sagging (laxity) Telangiectasia Wrinkles (coarse wrinkles) | Counted, then graded 0, 5, 30, or 75 according to frequency (SCINEXA) Presence or absence Graded 0-5 Graded 0-5 Graded 0-5 |
| Vierkötter et al., 2016^103^ | China; Germany; Japan | 2,326 females, 30-90 years, Asians and Caucasians | Pigmentation (pigment spots) | Graded 0-5 for severity; counted and graded 0-3 according to frequency category (SCINEXA) |
|  |  |  | Wrinkles (coarse wrinkles) | Graded 0-5 |
| Wang et al., 2009^104^ | China (Hangzhou) | 848 males and females, mean age 35.8, Asian | Wrinkles | Graded none, fine, moderate, severe |
| Warren et al., 1991^105^ | US (Arizona) | 41 females, 25-51 years, Caucasian | Wrinkles (furrows)  Perceived age | Measured according to method by Gartstein and Shaya (1986) Estimated in years |
| Yin et al., 2001^106^ | Japan | 83 males and females, 23-95 years, race NR | Wrinkles | Graded 1-6 (Daniell scale) |
| Yin et al., 2001^107^ | Japan | 63 males and females, mean age 67.5, race NR, | Skin surface microtopography | Quantified using NIH image processing system |
| Youn et al., 2003^108^ | South Korea | 186 females, 20-89 years, Asian | Wrinkles | Graded 0-7 (scale from Chung et al., 2001) |
| Zhao et al., 1998^109^ | China (north-eastern region) | 470 males and females, 10-80 years, Asian | Skin surface microtopography | Graded 1-6 (Beagley-Gibson scale) |

Supplementary Table 3. Skin aging definitions by studies from the literature search, where provided, paraphrased from the original text.

| **Study** | **Search** | **Definition of skin aging** |
| --- | --- | --- |
| Bhatt et al., 2019^9^ | Primary | Both intrinsic and extrinsic factors determine the skin aging process. Intrinsic aging or chronological aging is unavoidable, whereas extrinsic skin aging is dependent on environmental and behavioral factors. |
| Ekiz et al., 2012^20^ | Primary | Cutaneous ageing is a continuous process, with intrinsic factors determining which extrinsic factors (chronic sun exposure and other environmental factors, particularly smoking) have the greatest influence. The cutaneous changes that occur with age lead to a gradual physiological decline. |
| Green et al., 2011^40^ | Primary | The severity of skin aging at any particular age is mostly a function of the degree of skin photoaging. Superimposed on the thinning and fine wrinkling of chronological aging, photoaging causes skin dryness, irregular pigmentation and coarse wrinkles due to the destruction of skin elasticity by cumulative sun exposure. |
| Green, 1991^41^ | Primary | Degeneration due to sun exposure rather than simply to the passage of time is known as premature ageing of the skin or photoageing. |
| Hamer et al., 2017^44^ | Primary | Skin aging is an ongoing process associated with declined skin function and changes in its appearance. ... Both intrinsic and extrinsic factors contribute to skin aging; smoking and UV radiation are the most well-known extrinsic risk factors. |
| Latreille et al., 2013^62^ | Primary | Skin aging results from a combination of intrinsic and extrinsic aging factors. Intrinsic aging is an ineluctable process due to the natural degeneration of cell functioning that occurs with age and is genetically determined. … On the contrary, extrinsic aging is dependent on environmental and behavioral factors and can be prevented. |
| Leung & Harvey, 2002^63^ | Primary | There are two independent processes governing skin ageing: intrinsic and extrinsic ageing. Intrinsic ageing is the slow irreversible degeneration of tissue that affects almost all body organs. Extrinsic ageing (often termed photoageing) is widely thought to be due primarily to exposure to the ultraviolet (UV) radiation of the sun. |
| Peng et al., 2017^83^ | Primary | Skin ageing includes extrinsic and intrinsic ageing. |
| Raitio et al., 2004^88^ | Primary | Skin ageing is due to intrinsic ageing, where genetic factors play a major role, and extrinsic ageing, in which sun exposure is a well known factor, with distinct clinical and histological features. |
| Vierkötter et al., 2016^103^ | Primary | Ageing of the skin is influenced by two separate processes. The general ageing process, which is genetically determined and occurs over time alone, is called the intrinsic skin ageing process, whereas the skin ageing process induced by environmental factors is termed the extrinsic skin ageing process. |
| Youn et al., 2003^108^ | Primary | The ageing process of the skin can be divided into intrinsic ageing and photo-ageing. Damage to human skin as a result of repeated exposure to ultraviolet (UV) radiation from the sun (photo-ageing) and damage occurring because of the passage of time (chronologic ageing) are considered distinct entities rather than similar skin ageing processes. |
| Battistutta et al., 2006^7^ | Secondary | Skin photoaging is a complex process, comprising clinical, cellular, histological and immunological changes which reflect chronological ageing as well as damage directly attributable to ultraviolet radiation (UVR). |
| Buendía-Eisman et al., 2020^8^ | Secondary | *Cited from Krutmann et al., 2017*: ‘The skin ageing exposome consists of external and internal factors and their interactions, affecting a human individual from conception to death as well as the response of the human body to these factors that lead to biological and clinical signs of skin aging.’ |
| Castelo-Branco et al., 1998^10^ | Secondary | The normal aging process of the skin includes gradual thinning, atrophy, dryness, skin fragility and wrinkling. |
| Cosgrove et al., 2007^14^ | Secondary | Skin aging is a continuous process that is heavily determined by the combined influences arising from intrinsic aging, the environment (eg, sun exposure), and lifestyle factors [eg, cigarette smoking, low body mass index (BMI; in kg/m2), and menopausal status]. |
| Ding et al., 2017^17^ | Secondary | Skin aging is caused by both intrinsic and extrinsic factors. Extrinsic skin aging is known to be affected by various environmental factors including sun exposure, tobacco smoking, and air pollution. |
| Elfakir et al., 2010^21^ | Secondary | Aging of the skin is influenced both by intrinsic factors, such as chronological age, and by extrinsic or environmental factors, such as chronic UV exposure and smoking. Skin photoaging is defined as premature aging of the skin due to chronic sun exposure and presents characteristic morphological changes to both the epidermal and the dermal compartments. |
| Eun, 2001^24^ | Secondary | Skin aging can be divided into two basic processes. One is photoaging, which indicates premature skin aging in chronically photodamaged skin. |
| Flament et al., 2013^27^ | Secondary | *Described implicitly as the* 'result of several concomitant processes'*, with both chronological aging and photoaging contributing to clinical effects on the skin.* |
| Flament et al., 2015^28^ | Secondary | … overall aging process is the combined effects of chronological (intrinsic) and sun-induced (extrinsic) damages … |
| Goodman et al., 2018^38^ | Secondary | The appearance and structure of skin changes with age due to intrinsic (chronological) processes, extrinsic factors that include sun and UV exposure, gravity, pollution, and lifestyle factors such as diet, smoking, illness or stress. Solar UV irradiation is the primary extrinsic factor causing human skin ageing. The cumulative process of photoageing depends primarily on the degree of sun exposure and amount of skin pigment. |
| Hillebrand et al., 2001^46^ | Secondary | Skin photodamage or ‘photoaging’ is the superimposition of chronic sun damage (extrinsic aging) on top of intrinsic aging and is marked by an acceleration in the appearance of fine and coarse wrinkling, mottled pigmentation and lentigenes, and skin sagging. |
| Martires et al., 2009^68^ | Secondary | Aging is attributed to both intrinsic and extrinsic processes. Photoaging - the most recognised form of extrinsic aging of skin - describes changes brought about by long-term sun exposure, resulting in photodamage. Photodamage, therefore, refers to the physical and morphologic alterations secondary to solar UV exposure and is the main component of photoaging. |
| Qiu et al., 2011^86^ | Secondary | Internally, it undergoes progressive alterations, over decades, resulting from a regular decline in many biological functions, globally defined as the aging process, including intrinsic (chronological) and sun-induced, otherwise known as photo-ageing. These, combined, lead to slow and progressive changes in both skin functions and structures, ultimately altering physical appearance, most of the time assessed on the face by oneself and others. |
| Raduan et al., 2008^87^ | Secondary | Cutaneous ageing is a continuous process involving intrinsic ageing (a universal and inevitable alteration attributed to the passage of time) and extrinsic ageing (the superposition over intrinsic ageing of changes attributed to chronic solar exposure and other environmental factors, among them, smoking). |
| Takahashi et al., 2017^97^ | Secondary | Skin aging occurs intrinsically chronological effect and influenced by environmental and genetic factors. |
| Vierkoetter et al., 2009^101^ | Secondary | Skin ageing is the consequence of intrinsic and extrinsic factors. Intrinsic or chronological skin ageing and extrinsic skin ageing, which is primarily due to chronic exposure to ultraviolet (UV) radiation and therefore is also called photoageing, can be distinguished histologically and clinically, involve distinct pathogenetic causes and mechanisms and their prevention is based on different principles. |
| Vierkoetter et al., 2010^102^ | Secondary | Aging results from the combined action of intrinsic and extrinsic factors. |
| Yin et al., 2001^107^ | Secondary | Premature skin aging is the result of programmed senescence and prolonged environmental injury to skin. |


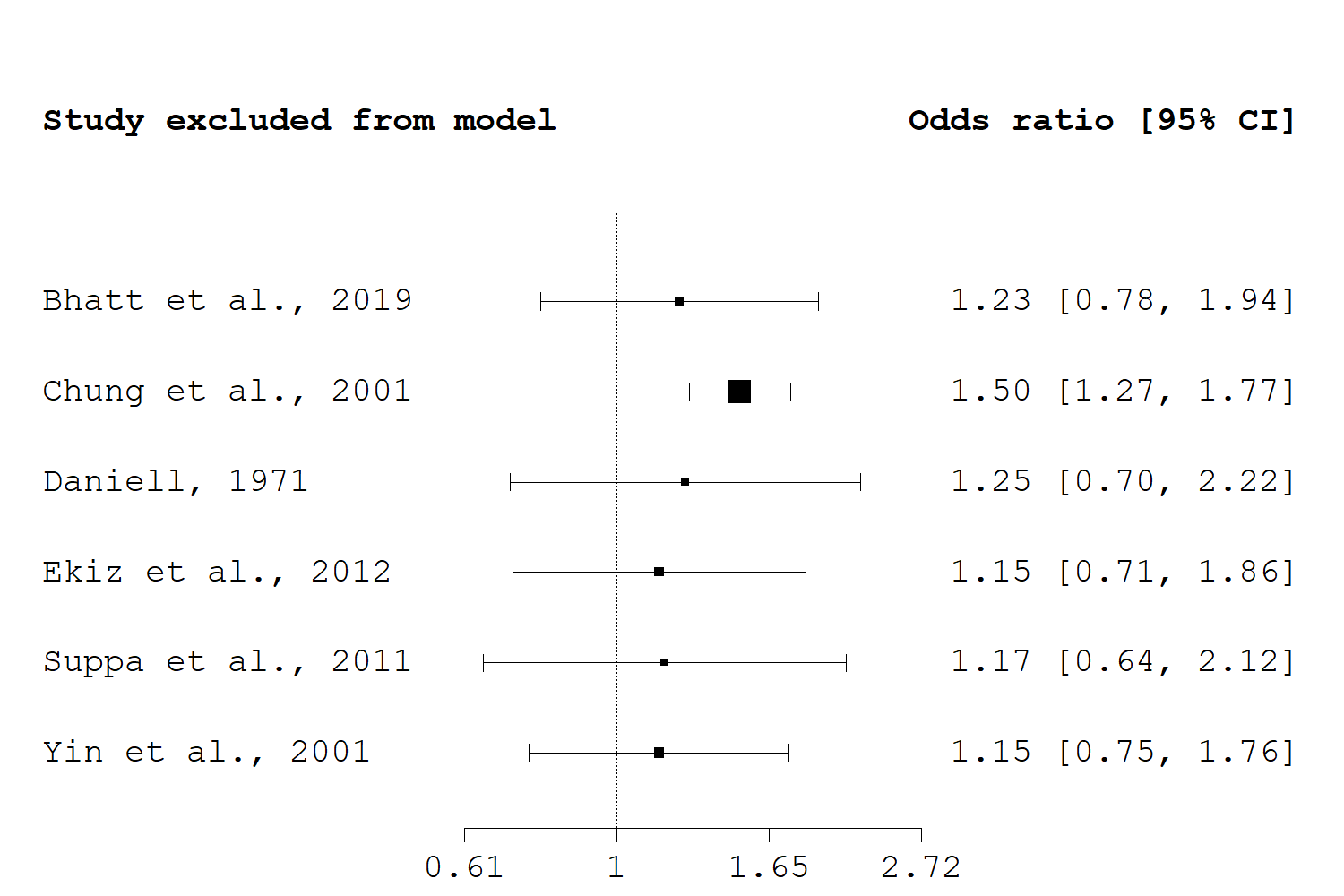


Supplementary Figure 1. Sensitivity analysis for meta-analysis of wrinkling and male gender.

Supplementary Table 4. Summary of associations for skin aging signs and air pollutant exposure.

| **Pollutant/exposure** | **Subject characteristics** | **Outcome** | **Association/Effect size, if reported** | **Study** |
| --- | --- | --- | --- | --- |
| AQI | 201 males, 20-60 years, Asian | Greater perceived age Pigmentation Sagging Telangiectasia Wrinkling | S S (20-39 years and 50-59 years only) S (20-29 years only) S (20-29 years and 40-59 years only) S | Flament et al., 2019^30^ |
|  | 204 females, 25-45 years, Asian | Pigmentation Wrinkling | S (30 years and above) S (30 years and above) | Flament et al., 2018^29^ |
| CO | 188 males and females, 21-66 years | Pigmentation Wrinkling | spearman's r: 1.15, SE: 1.53, p=0.453 spearman's r: 2.81, SE: 2.25, p=0.212 | Park et al., 2019^82^ |
| Contact with fossil fuels | 400 females, 40-90 years, Asian | Lentigines (cheek) | OR: 2.358, 95% CI: 1.152-4.825 | Peng et al., 2017^83^ |
| Cooking with solid fuels | 1129 females, 30-90 years, Asian | Laxity (cheeks) Laxity (eyelids) Lentigines Wrinkling (crows feet) Wrinkling (fine, hand dorsum) Wrinkling (forehead) Wrinkling (frownlines) Wrinkling (nasolabial fold) Wrinkling (upper lip) | AMR: 1.06, 95% CI: 1.01-1.11 AMR: 1.06, 95% CI: 1.02-1.11 NS AMR: 1.06, 95% CI: 1.02-1.11 OR: 1.74, 95% CI: 1.2-2.55 AMR: 1.09, 95% CI: 1.05-1.14 AMR: 1.05, 95% CI: 1.01-1.11 AMR: 1.08, 95% CI: 1.04-1.11 AMR: 1.07, 95% CI: 1.01-1.13 | Li et al., 2015^64^ |
| Distance <=100 m from a busy road | 400 females, 68-79 years, Caucasian (SALIA study) | Skin aging signs in SCINEXA scale | NS | Vierkoetter et al., 2010^102^ |
| NO_2_ | 1072 males and females, 27-90 years, Asian (Taizhou) | Lentigines (cheek) | GMR: 1.11, 95% CI: 1.03-1.20 | Hüls et al., 2016^48^ |
|  | 806 females, 66-79 years, Caucasian (SALIA) | Lentigines (cheek) | S | Hüls et al., 2016^48^ |
| O_3_ | 188 males and females, 21-66 years | Pigmentation  Wrinkling | spearman's r: 0.02, SE: 0.02, p=0.34 spearman's r: -0.04, SE: 0.04, p=0.278 | Park et al., 2019^82^ |
|  | 2013 males and females, 20-84 years (SALIA and BASE-II) | Lentigines Wrinkling (forehead) | NS % change: 8.34, 95% CI: 2.01-14.67 | Fuks et al., 2019^34^ |
| Occupations exposed to toxic and harmful substances | 400 females, 40-90 years, Asian | Lentigines Seborrheic keratosis | NS NS | Peng et al., 2017^83^ |
| PM10 | 188 males and females, 21-66 years | Pigmentation  Wrinkling | spearman's r: 0.02, SE: 0.02, p=0.164 spearman's r: 0.04, SE: 0.03, p=0.109 | Park et al., 2019^82^ |
|  | 400 females, 68-79 years, Caucasian (SALIA study) | Pigmentation (cheeks) Wrinkling (nasolabial fold) | MR/OR: 1.08, 95% CI: 1.01-1.15 MR/OR: 1.01, 95% CI: 1.01-1.02 | Vierkoetter et al., 2010^102^ |
| PM2.5 | 188 males and females, 21-66 years | Pigmentation  Wrinkling | spearman's r: -0.01, SE: 0.03, p=0.657 spearman's r: -0.06, SE: 0.04, p=0.192 | Park et al., 2019^82^ |
|  | 799 females, 69-79 years, Caucasian (SALIA) | Lentigines (cheek) | S | Hüls et al., 2019^49^ |
|  | 1877 males and females, 35-89 years, Asian (Taizhou) | Cutis rhomboidalis nuchae Laxity (eyelids) Lentigines (forehead) Wrinkling (fine, hand dorsum) Wrinkling (forehead) Wrinkling (under eyes) Wrinkling (upper lip) | OR: 1.346, 95% CI: 1.086-1.669 AMR: 1.032, 95% CI: 1.012-1.052 AMR: 1.079, 95% CI: 1.017-1.141 OR: 1.4, 95% CI: 1.017-1.927 AMR: 1.033, 95% CI: 1.004-1.062 AMR: 1.034, 95% CI: 1.004-1.064 AMR: 1.094, 95% CI: 1.049-1.138 | Ding et al., 2017^17^ |
| PM2.5 (low vs high exposure) | 400 females, 40-90 years, Asian | Lentigines (cheek) Lentigines (hand dorsum) | OR: 0.403, 95% CI: 0.239-0.679 OR: 0.263, 95% CI: 0.156-0.441 | Peng et al., 2017^83^ |
| Second-hand smoking | 400 females, 40-90 years, Asian | Lentigines (cheek) Seborrheic keratosis (hand dorsum) | OR: 1.784, 95% CI: 1.145-2.781 OR: 2.341, 95% CI: 1.288-4.254 | Peng et al., 2017^83^ |
| SO_2_ | 188 males and females, 21-66 years | Pigmentation Wrinkling | spearman's r: -0.43, SE: 0.19, p=0.021 spearman's r: -0.31, SE: 0.26, p=0.235 | Park et al., 2019^82^ |
| Soot | 400 females, 68-79 years, Caucasian (SALIA study) | Pigmentation (cheeks) Pigmentation (forehead) Wrinkling (nasolabial fold) | MR/OR: 1.22, 95% CI: 1.03-1.45 MR/OR: 1.2, 95% CI: 1.03-1.40 MR/OR: 1.04, 95% CI: 1.01-1.06 | Vierkoetter et al., 2010^102^ |
| Traffic-associated particles | 400 females, 68-79 years, Caucasian (SALIA study) | Pigmentation (cheeks) Pigmentation (forehead) Wrinkling (nasolabial fold) | MR/OR: 1.17, 95% CI: 1.08-1.27 MR/OR: 1.16, 95% CI: 1.06-1.27 MR/OR: 1.03, 95% CI: 1.01-1.04 | Vierkoetter et al., 2010^102^ |

Supplementary Table 5. Summary of associations for skin aging signs and nutritional intake.

| **Exposure** | | **Outcome** | **Significant association** | **Study** |
| --- | --- | --- | --- | --- |
| Alcohol | | Wrinkling Pigmentation | none none | Park et al., 2019^82^ |
|  |  | Skin surface microtopography | none | Purba et al., 2001^85^ |
| Carbohydrate (50g Increase) | | Thinning | increased likelihood | Cosgrove et al., 2007^14^ |
| Dairy | | Skin surface microtopography | increase | Purba et al., 2001^85^ |
| Fatty Acids | Linoleic Acid | Dryness Thinning | lower likelihood lower likelihood | Cosgrove et al., 2007^14^ |
|  | ALA DPA EPA PUFA | Photoaging Photoaging Photoaging Photoaging | decrease (men only) decrease (women with highest intakes only) decrease (women with highest intakes only) decrease (women with highest intakes only) | Latreille et al., 2013^62^ |
|  | MUFA PUFA | Wrinkling Wrinkling | none none | Nagata et al., 2010^77^ |
|  | MUFA | Skin surface microtopography | decrease | Purba et al., 2001^85^ |
| Fat | Fat (17g Increase) | Wrinkling | increased likelihood | Cosgrove et al., 2007^14^ |
|  | Fat Saturated Fat | Wrinkling Wrinkling | none increase (after adjustment for green & yellow vegetable intake) | Nagata et al., 2010^77^ |
|  | Fat Oils And Fats | Skin surface microtopography Skin surface microtopography | decrease decrease | Purba et al., 2001^85^ |
| Fruit Dutch Healthy Diet Index Unhealthy Diet | | Wrinkling Wrinkling Wrinkling | decrease (women only) decrease (women only) increase (women only) | Mekic et al., 2019^70^ |
| Instant Food Intake | | Wrinkling Pigmentation | none none | Park et al., 2019^82^ |
| Micronutrients | Zinc | Wrinkling | none | Nagata et al., 2010^77^ |
|  | Calcium Iron Magnesium Phosphorus Zinc | Skin surface microtopography Skin surface microtopography Skin surface microtopography Skin surface microtopography Skin surface microtopography | decrease decrease decrease decrease decrease | Purba et al., 2001^85^ |
| Seafood | Fish & Shell Fish | Wrinkling | none | Nagata et al., 2010^77^ |
|  | Fish | Skin surface microtopography | decrease | Purba et al., 2001^85^ |
| Sugar Products | | Skin surface microtopography | increase | Purba et al., 2001^85^ |
| Vegetables | Green & Yellow Vegetables | Wrinkling | decrease | Nagata et al., 2010^77^ |
|  | Legumes Vegetables | Skin surface microtopography Skin surface microtopography | decrease decrease | Purba et al., 2001^85^ |
| Vitamins | Vitamin C | Dryness Wrinkling | lower likelihood lower likelihood | Cosgrove et al., 2007^14^ |
|  | Vitamin C | Wrinkling | none | Nagata et al., 2010^77^ |
|  | Vitamin C Retinol | Skin surface microtopography Skin surface microtopography | decrease decrease | Purba et al., 2001^85^ |

Supplementary Table 6. Summary of associations for skin aging signs and sun exposure.

| **Outcome** | **N** | **Exposure** | **Effect size, if reported** | **Significance** | **Study** |
| --- | --- | --- | --- | --- | --- |
| Dryness | 3808 | increase | NR | S (p<0.001) | Cosgrove et al., 2007^14^ |
| Elastosis | 20295 | high exposure | NR | S (p<0.001), Caucasian males and females | Engel et al., 1988^22^ |
|  | 956 | lifetime, >40 h x1000 vs 8-20 h x1000 lifetime, 20-30 h x1000 vs 8-20 h x1000 lifetime, 30-40 h x1000 vs 8-20 h x1000 | OR: 2.8, 95% CI: 1.3-6.1 OR: 1.3, 95% CI: 0.7-2.4 OR: 2.3, 95% CI: 1.2-4.4 | S NS S | Kennedy et al., 2003^54^ |
|  | 560 | increase (lifetime h) | OR: 1.000028, 95% CI: 0.999931-1.000125 | NS | Leung & Harvey, 2002^63^ |
| Ephelides | 961 | increase (h/y) increase (lifetime h) | NR NR | S (p=0.01) S (p<0.0001) | Bastiaens et al., 2004^6^ |
|  | 20295 | high exposure | NR | S (p<0.001), Caucasian males and females | Engel et al., 1988^22^ |
|  | 523 | High score values vs Low score values Medium score values vs Low score values | OR: 1.10, 95% CI: 0.71-1.70 OR: 1.32, 95% CI: 0.86-2.04 | NS NS | Ezzedine et al., 2013^25^ |
| Lentigines | 961 | increase (h/y) increase (lifetime h) | NR NR | NS S (p<0.0001) | Bastiaens et al., 2004^6^ |
|  | 20295 | high exposure | NR | S (p<0.01), Caucasian females | Engel et al., 1988^22^ |
|  | 523 | High score values vs Low score values Medium score values vs Low score values | OR: 2.12, 95% CI: 1.36-3.31 OR: 1.79, 95% CI: 1.15-2.79 | S S | Ezzedine et al., 2013^25^ |
| Perceived age | 196 | increase | NR | S | Guyuron et al., 2009^43^ |
|  | 1826 | increase | NR | S, males only | Rexbye et al., 2006^89^ |
|  | 41 | increase | NR | S (45-51 years) | Warren et al., 199^105^ |
| Perceived age (decrease) | 220 | less time spent in the sun | NR | S | Mayes et al., 2010^69^ |
| Photoaging | 75 | increase | linear regression B: -0.388, p=0.063 | NS | Chien et al., 2018^12^ |
|  | 530 | High vs Low/moderate | OR: 1.38, 95% CI: 0.91-2.11 | NS | Elfakir et al., 2010^21^ |
|  | 20295 | high exposure | NR | S, Caucasian males and females, Black males | Engel et al., 1988^22^ |
|  | 6630 | Mild vs None, females Mild vs None, males Moderate vs None, females Moderate vs None, males Severe vs None, females Severe vs None, males | OR: 0.9, 95% CI: 0.5-1.8 OR: 0.6, 95% CI: 0.4-1.3 OR: 1.0, 95% CI: 0.5-1.9 OR: 0.7, 95% CI: 0.4-1.4 OR: 1.1, 95% CI: 0.5-2.2 OR: 0.8, 95% CI: 0.4-1.6 | NS NS NS NS NS NS | Malvy et al., 2000^67^ |
|  | 120 | sun exposure in automobile | NR | Time spent as automobile driver was significantly associated with higher photodamage score of the left face, which was exposed to the sun when driving (p=0.015) | Singer et al., 1994^93^ |
| Pigmentation | 20295 | high exposure | NR | S | Engel et al., 1988^22^ |
|  | 180 | increase | r=0.0003, SE: 0.00007, p<0.01 | S | Kimlin & Guo, 2012^58^ |
|  | 298 | increase | NR | S | Flament et al., 2013^27^ |
|  | 301 | increase | NR | S | Flament et al., 2015^28^ |
|  | 1011 | increase | NR | S | Flament et al., 2019^31^ |
|  | 261 | increase | NR | S (above 31 years only) | Flament et al., 2019^32^ |
| Pores | 301 | increase | NR | NS | Flament et al., 2015^28^ |
|  | 261 | increase | NR | no consistent trend across age groups | Flament et al., 2019^32^ |
| Sagging | 298 | increase | NR | NS | Flament et al., 2013^27^ |
|  | 301 | increase | NR | S | Flament et al., 2015^28^ |
|  | 1011 | increase | NR | S (above 31 years only) | Flament et al., 2019^31^ |
|  | 261 | increase | NR | S (above 60 years only) | Flament et al., 2019^32^ |
| Seborrheic keratosis | 303 | >6 h/d vs <3h/d 3-6 h/d vs <3h/d | POR: 2.28, 95% CI: 1.19-4.38 POR: 1.35, 95% CI: 0.68-2.68 | S NS | Kwon et al., 2003^61^ |
| Skin surface microtopography | 691 | hours, 2nd tertile vs 1st tertile hours, 3rd tertile vs 1st tertile | OR: 2.1, 95% CI: 1.3-3.3 OR: 4.3, 95% CI: 2.2-8.3 | S S | Gao et al., 2013^35^ |
|  | 470 | high vs low high vs low | OR: 2.08, 95% CI: 1.41-3.07 OR: 2.09, 95% CI: 1.36-3.21 | S S | Zhao et al., 1998^109^ |
| Solar keratosis | 20295 | high exposure | NR | S (p<0.001), Caucasian males and females | Engel et al., 1988^22^ |
|  | 966 | lifetime, >=40000 h vs 8932 - 19999 h lifetime, 20000 - 29999 h vs 8932 - 19999 h lifetime, 30000 - 39999 h vs 8932 - 19999 h | OR: 4.5, 95% CI: 1.9-10.5 OR: 1.60, 95% CI: 0.79-3.00 OR: 1.80, 95% CI: 0.81-3.80 | S NS NS | Kennedy et al., 2003^55^ |
| Telangiectasia | 20295 | high exposure | NR | S (p<0.001), Caucasian males and females | Engel et al., 1988^22^ |
|  | 298 | increase | NR | NS | Flament et al., 2013^27^ |
|  | 1011 | increase | NR | does not discriminate between high and low sun exposures consistently across age classes | Flament et al., 2019^31^ |
|  | 261 | increase | NR | S (31-40 years and above 51 years only) | Flament et al., 2019^32^ |
|  | 956 | lifetime, >40 h x1000 vs 8-20 h x1000, female lifetime, >40 h x1000 vs 8-20 h x1000, male lifetime, 20-30 h x1000 vs 8-20 h x1000, female lifetime, 20-30 h x1000 vs 8-20 h x1000, male lifetime, 30-40 h x1000 vs 8-20 h x1000, female lifetime, 30-40 h x1000 vs 8-20 h x1000, male | OR: 1.9, 95% CI: 0.64-5.9 OR: 4.8, 95% CI: 1.2-18.7 OR: 1.4, 95% CI: 0.7-2.8 OR: 2, 95% CI: 0.6-6.7 OR: 1.9, 95% CI: 0.81-4.7 OR: 1.4, 95% CI: 0.37-5.7 | NS S NS NS NS NS | Kennedy et al., 2003^54^ |
| Telangiectasia | 1342 | increase (daily hours) | OR: 1.12, 95% CI: 1.02-1.24 | S | Suppa et al., 2011^96^ |
| Thinning | 3808 | increase | NR | S (p<0.001) | Cosgrove et al., 2007^14^ |
| Wrinkling | 650 | increase | NR | NS | Allen et al., 1973^3^ |
|  | 110 | >1h/d vs <1h/d | OR: 2.478, 95% CI: 1.093-5.62 | S | Bhatt et al., 2019^9^ |
|  | 370 | >4 h/d vs <2 h/d 2-4 h/d vs <2 h/d | RR: 2.26, 95% CI: 1.70-4.24 RR: 1.72, 95% CI: 0.87-3.19 | S NS | Castelo-Branco et al., 1998^10^ |
|  | 407 | >=5h/d vs 1-2h/d 3-4h/d vs 1-2h/d | OR: 4.85, 95% CI: 2.35-10.17 OR: 0.84, 95% CI: 0.31-2.24 | S NS | Chung et al., 2001^13^ |
|  | 3808 | increase | NR | S (p<0.001) | Cosgrove et al., 2007^14^ |
|  | 574 | >10 h/w vs <=10 h/w | OR: 1.12, 95% CI: 0.79-1.60 | NS | Ekiz et al., 2012^20^ |
|  | 1136 | >2 h/d vs <1 h/d, females 1-2 h/d vs <1 h/d, females daily, any exposure, males | OR: 2.3, 95% CI: 1.2-4.3 OR: 1.5, 95% CI: 0.8-2.5 NR | S NS NS | Ernster et al., 1995^23^ |
|  | 298 | increase | NR | S | Flament et al., 2013^27^ |
|  | 301 | increase | NR | S | Flament et al., 2015^28^ |
|  | 1011 | increase | NR | S | Flament et al., 2019^31^ |
|  | 261 | increase | NR | S (above 41 years only) | Flament et al., 2019^32^ |
|  | 82 | daily hours | r=0.02, p=0.87 | NS | Helfrich et al., 2007^45^ |
|  | 132 | lifetime, >50,000 h vs <50,000 h | RR: 3.1, 95% CI: 1.2-7.1 | S | Kadunce et al., 1991^53^ |
|  | 180 | increase | r=0.003, SE: 0.00007, p<0.01 | S | Kimlin & Guo, 2012^58^ |
|  | 716 | increase (cumulative h) | spearman's r: 0·17, p<0.01 | S | Nagata et al., 2010^77^ |
|  | 301 | strong exposure, yes vs no | OR: 2.72, p-value: 0.016 | S | Raduan et al., 2008^87^ |
|  | 89 | increase | NR | NS | Raitio et al., 2004^88^ |
|  | 848 | increase | NR | S, especially for the higher age groups | Wang et al., 2009^104^ |
|  | 41 | increase | NR | S (45-51 years) | Warren et al., 1991^105^ |
|  | 83 | >=2 h/d vs <2 h/d | OR: 2.65, 95% CI: 1.00-7.00 | S | Yin et al., 2001^106^ |


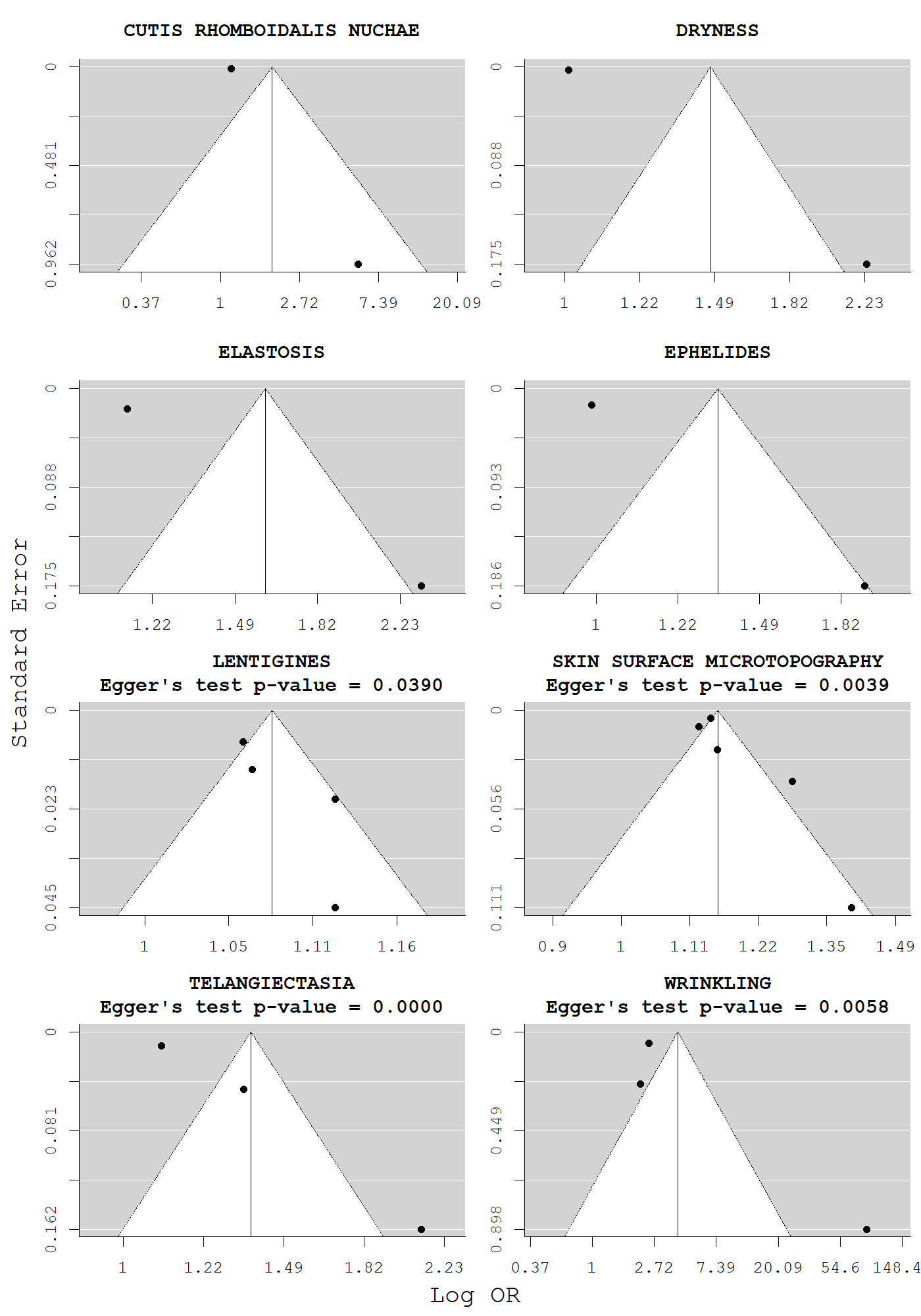


Supplementary Figure 2. Begg’s funnel plots and Egger’s test p-values for age and skin aging.


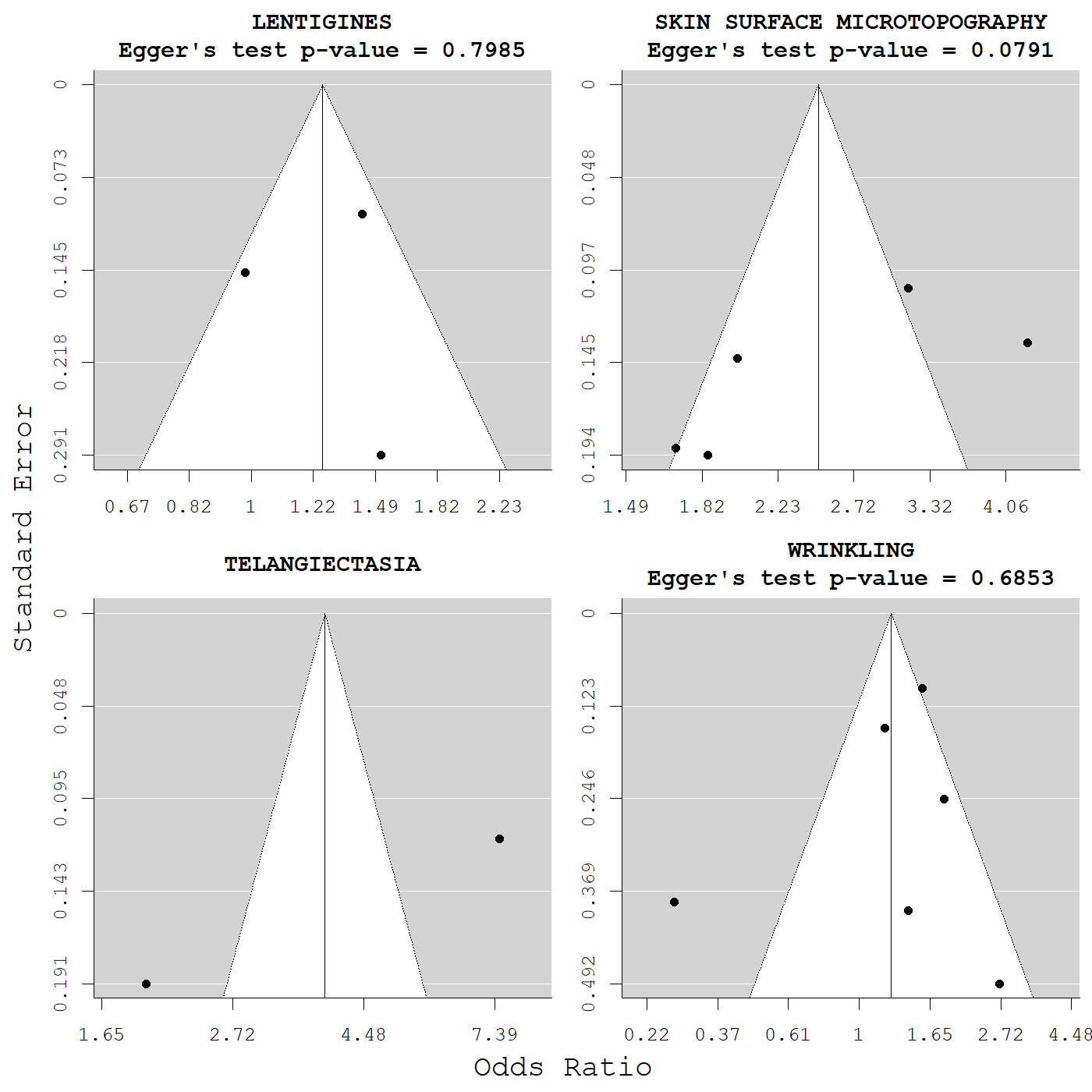


Supplementary Figure 3. Begg’s funnel plots and Egger’s test p-values for male gender (female gender as reference) and skin aging.


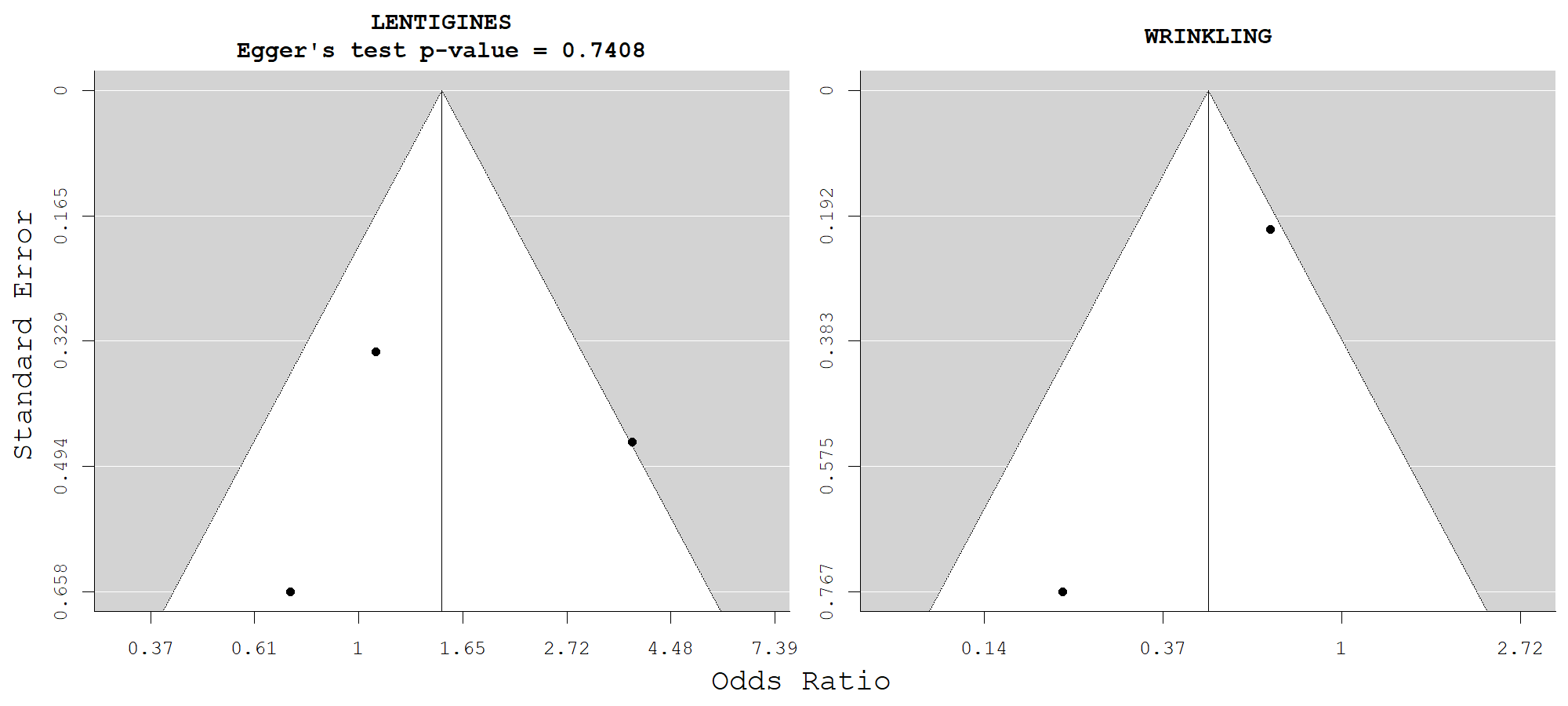


Supplementary Figure 4. Begg’s funnel plots and Egger’s test p-values for HRT us and skin aging.


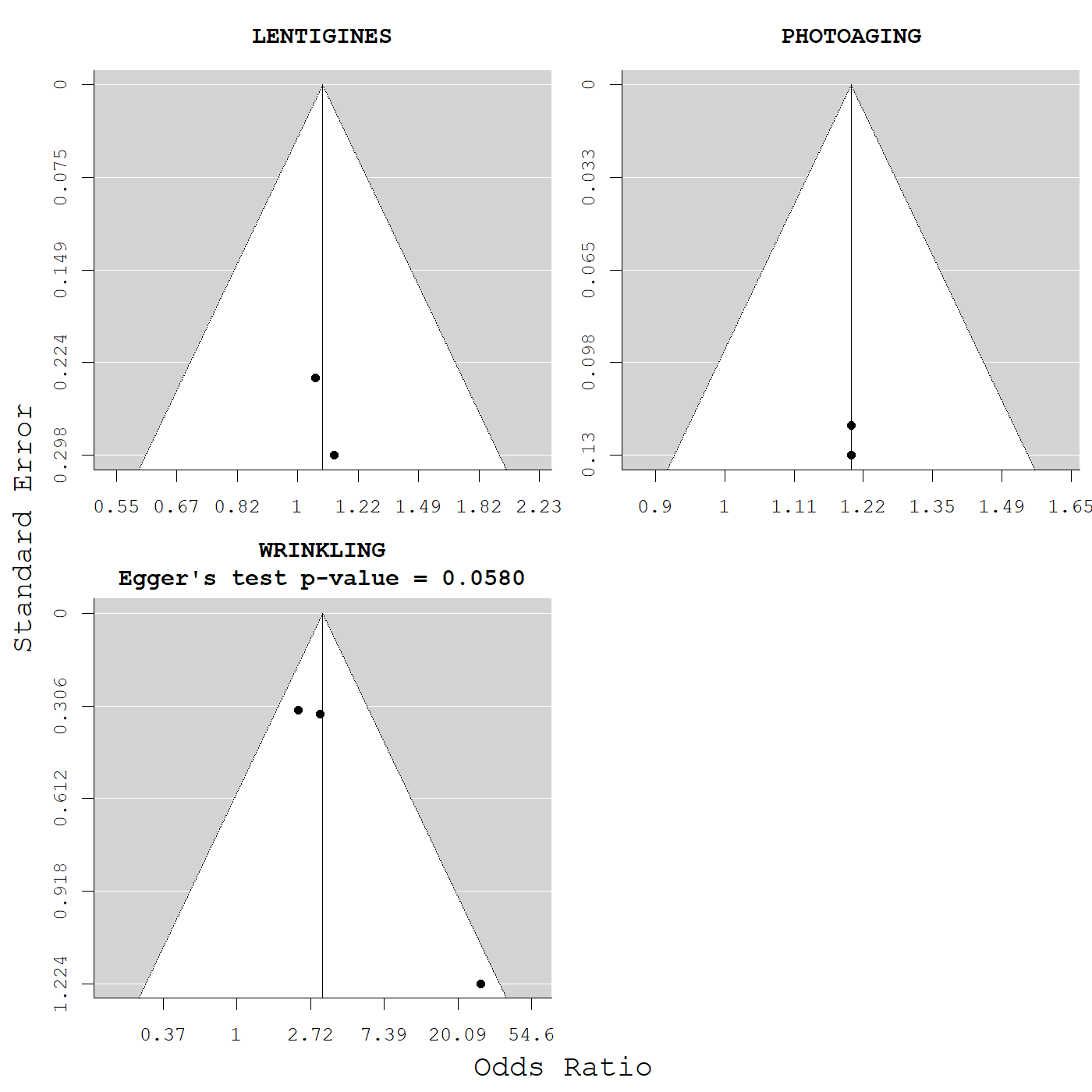


Supplementary Figure 5. Begg’s funnel plots and Egger’s test for current smoking and skin aging.


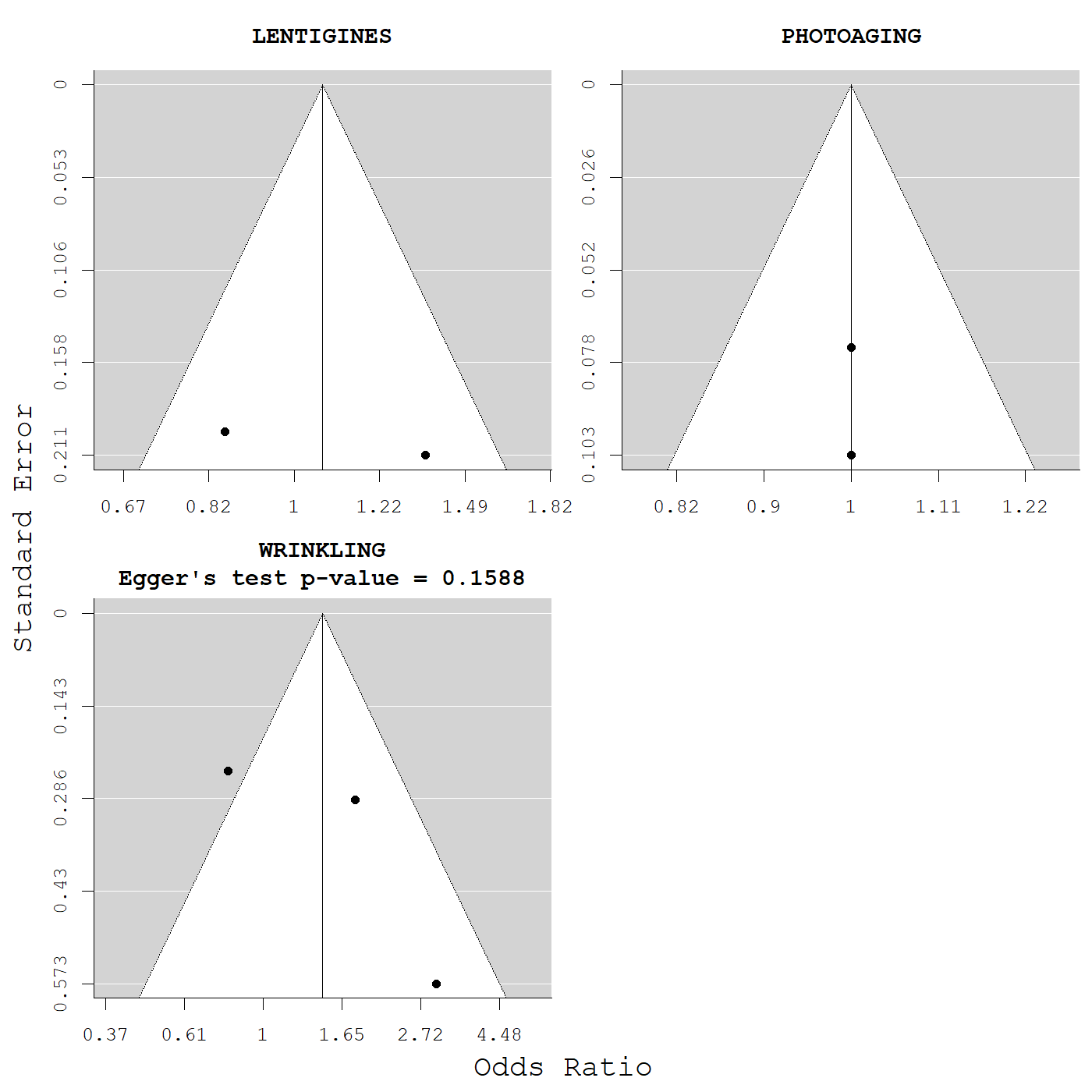


Supplementary Figure 6. Begg’s funnel plots and Egger’s test for former smoking and skin aging.


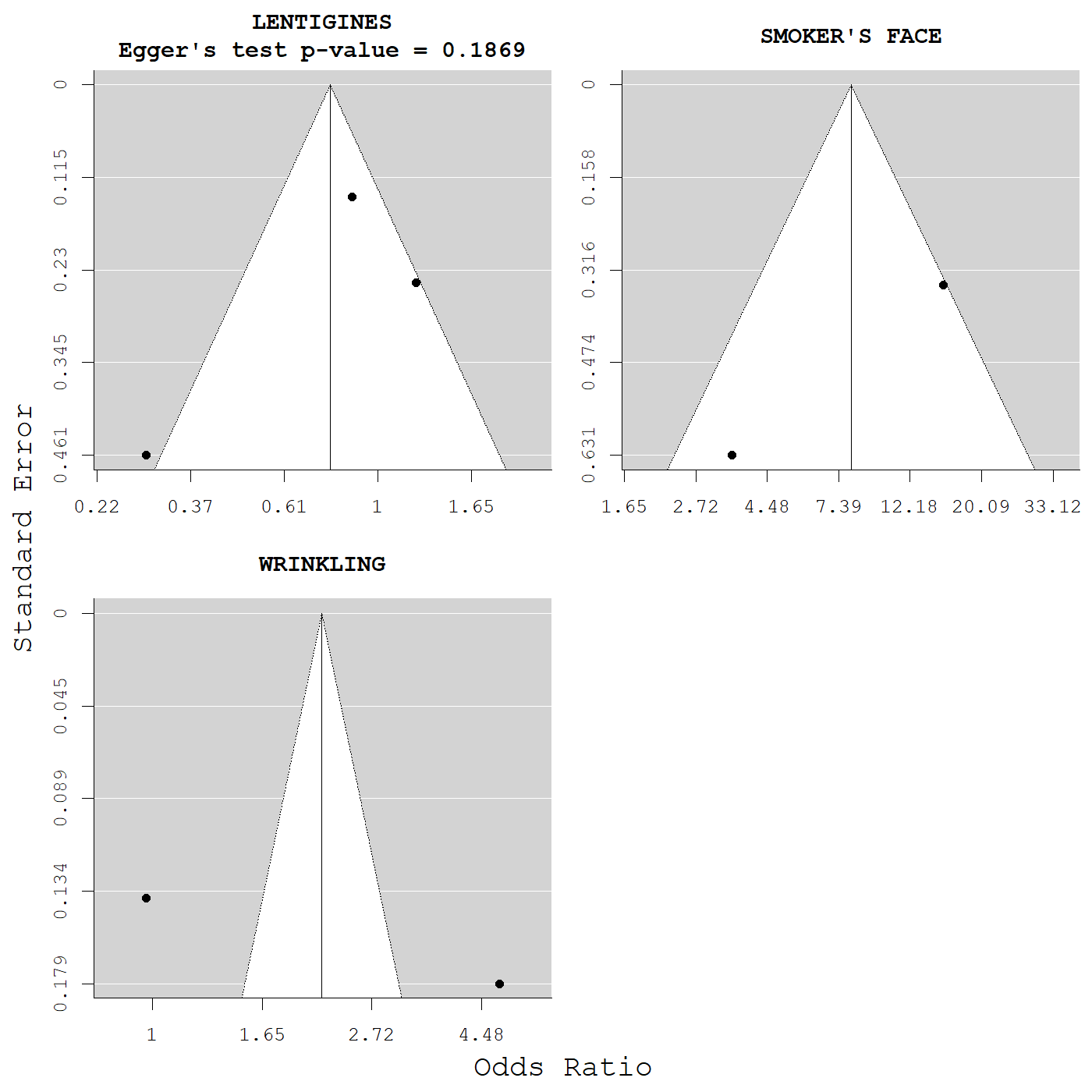


Supplementary Figure 7. Begg’s funnel plots and Egger’s test for former smoking and skin aging.


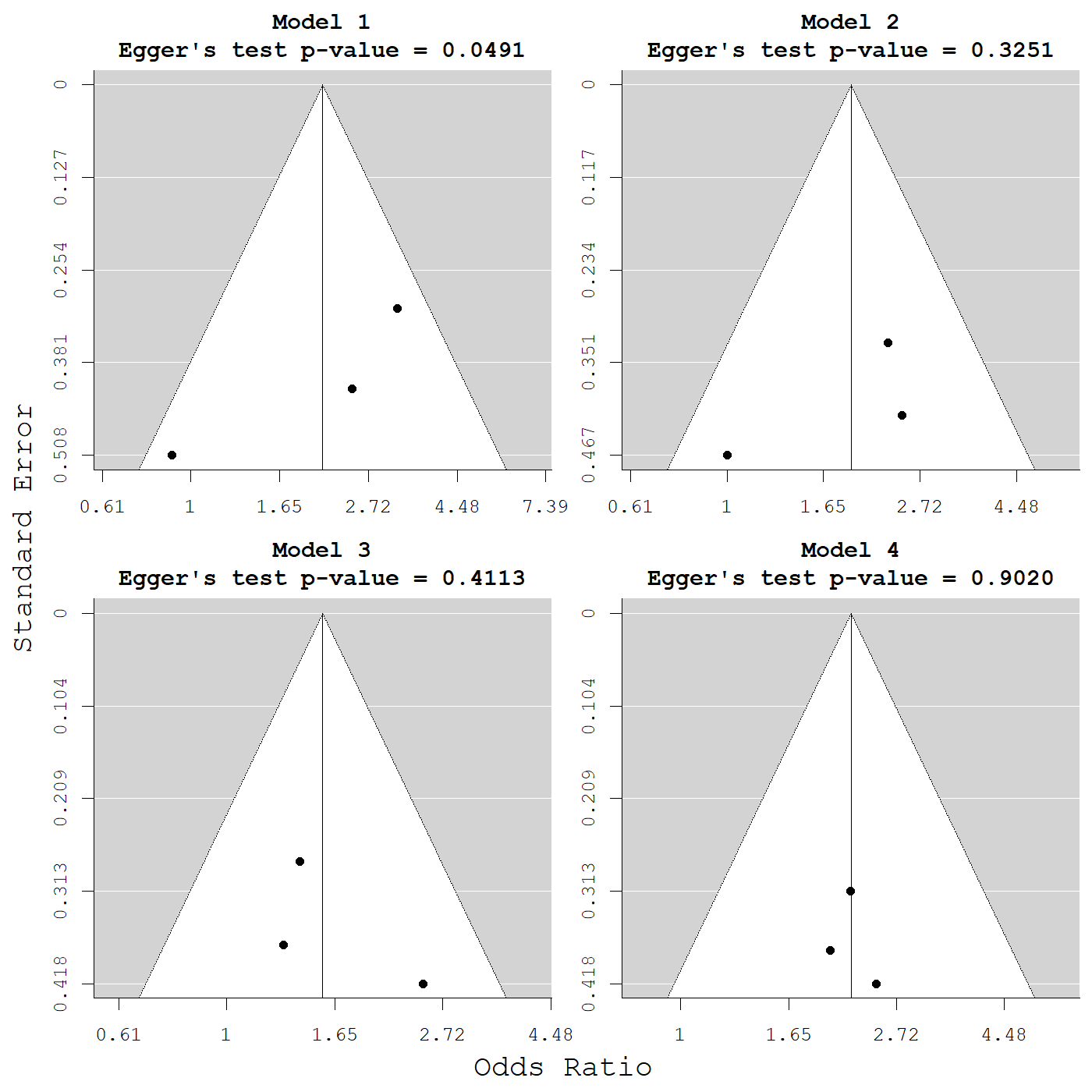


Supplementary Figure 8. Begg’s funnel plots and Egger’s test for more than 1h/d of sun exposure and wrinkling.


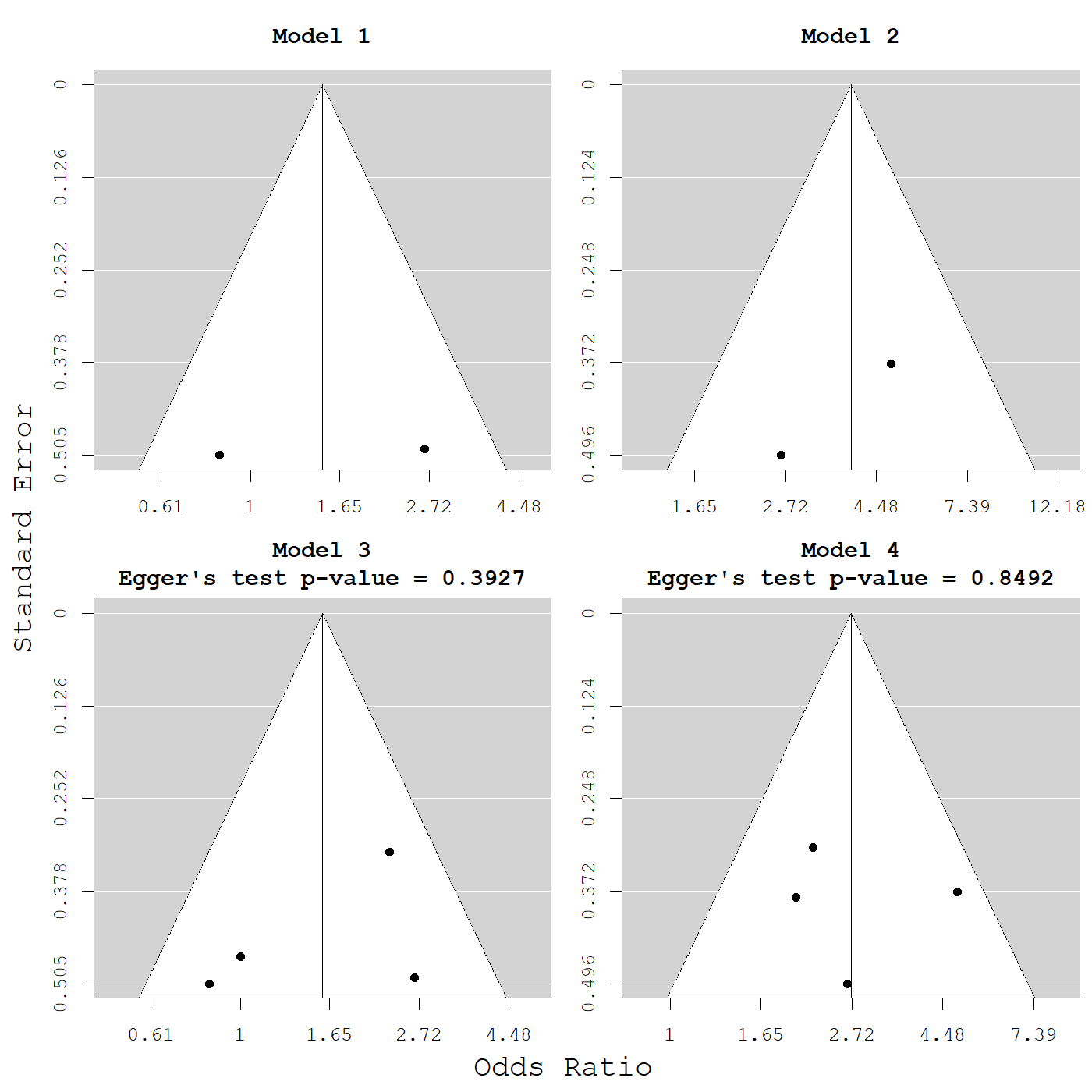


Supplementary Figure 9. Begg’s funnel plots and Egger’s test for more than 2h/d of sun exposure and wrinkling (compared with less than 2h/d of sun exposure).


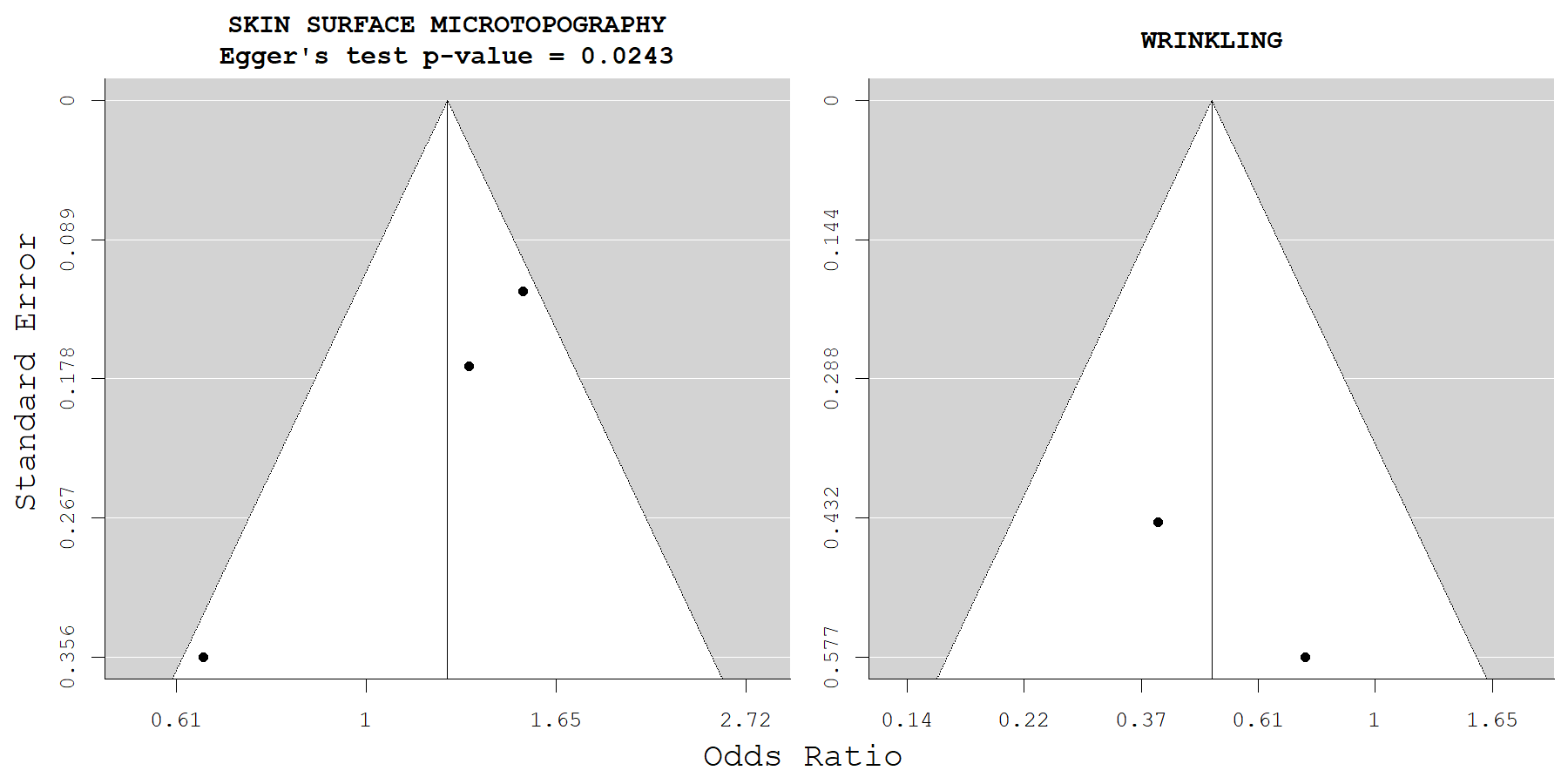


Supplementary Figure 10. Begg’s funnel plots and Egger’s test for sunscreen use and skin aging.

# **Supplementary References**

1 Aizen, E. & Gilhar, A. Smoking effect on skin wrinkling in the aged population. *International Journal of Dermatology* **40**, 431-433, doi:<https://doi.org/10.1046/j.1365-4362.2001.01238.x> (2001).

2 Akiba, S. *et al.* Influence of chronic UV exposure and lifestyle on facial skin photo-aging--results from a pilot study. *J Epidemiol* **9**, S136-142, doi:10.2188/jea.9.6sup_136 (1999).

3 Allen, H. B., Johnson, B. L. & Diamond, S. M. Smoker's wrinkles? *Jama* **225**, 1067-1069 (1973).

4 Asakura, K. *et al.* Lifestyle factors and visible skin aging in a population of Japanese elders. *J Epidemiol* **19**, 251-259, doi:10.2188/jea.je20090031 (2009).

5 Bastiaens, M. T., Westendorp, R. G., Vermeer, B. J. & Bavinck, J. N. Ephelides are more related to pigmentary constitutional host factors than solar lentigines. *Pigment Cell Res* **12**, 316-322, doi:10.1111/j.1600-0749.1999.tb00765.x (1999).

6 Bastiaens, M., Hoefnagel, J., Westendorp, R., Vermeer, B. J. & Bouwes Bavinck, J. N. Solar lentigines are strongly related to sun exposure in contrast to ephelides. *Pigment Cell Res* **17**, 225-229, doi:10.1111/j.1600-0749.2004.00131.x (2004).

7 Battistutta, D. *et al.* Skin surface topography grading is a valid measure of skin photoaging. *Photodermatology, Photoimmunology & Photomedicine* **22**, 39-45, doi:<https://doi.org/10.1111/j.1600-0781.2006.00194.x> (2006).

8 Buendia-Eisman, A., Prieto, L., Abarquero, M. & Arias-Santiago, S. Study of the Exposome Ageing-related Factors in the Spanish Population. *Acta Derm Venereol* **100**, adv00153, doi:10.2340/00015555-3500 (2020).

9 Bhatt, N., Agrawal, S. & Mehta, K. Risk factors and self-perception for facial aging among Nepalese population. *Journal of Cosmetic Dermatology* **18**, 1794-1799, doi:<https://doi.org/10.1111/jocd.12885> (2019).

10 Castelo-Branco, C., Figueras, F., Martínez de Osaba, M. J. & Vanrell, J. A. Facial wrinkling in postmenopausal women. Effects of smoking status and hormone replacement therapy. *Maturitas* **29**, 75-86, doi:10.1016/s0378-5122(97)00087-x (1998).

11 Chien, A. L. *et al.* Perioral wrinkles are associated with female gender, aging, and smoking: Development of a gender-specific photonumeric scale. *J Am Acad Dermatol* **74**, 924-930, doi:10.1016/j.jaad.2015.11.042 (2016).

12 Chien, A. L. *et al.* Effect of Age, Gender, and Sun Exposure on Ethnic Skin Photoaging: Evidence Gathered Using a New Photonumeric Scale. *J Natl Med Assoc* **110**, 176-181, doi:10.1016/j.jnma.2017.05.001 (2018).

13 Chung, J. H. *et al.* Cutaneous photodamage in Koreans: influence of sex, sun exposure, smoking, and skin color. *Arch Dermatol* **137**, 1043-1051 (2001).

14 Cosgrove, M. C., Franco, O. H., Granger, S. P., Murray, P. G. & Mayes, A. E. Dietary nutrient intakes and skin-aging appearance among middle-aged American women. *Am J Clin Nutr* **86**, 1225-1231, doi:10.1093/ajcn/86.4.1225 (2007).

15 Daniell, H. W. Smoker's wrinkles. A study in the epidemiology of "crow's feet". *Ann Intern Med* **75**, 873-880, doi:10.7326/0003-4819-75-6-873 (1971).

16 Derancourt, C. *et al.* Multiple large solar lentigos on the upper back as clinical markers of past severe sunburn: a case-control study. *Dermatology* **214**, 25-31, doi:10.1159/000096909 (2007).

17 Ding, A. *et al.* Indoor PM2.5 exposure affects skin aging manifestation in a Chinese population. *Sci Rep* **7**, 15329, doi:10.1038/s41598-017-15295-8 (2017).

18 Dobos, G. *et al.* Quantifying dyspigmentation in facial skin ageing: an explorative study. *Int J Cosmet Sci* **37**, 542-549, doi:10.1111/ics.12233 (2015).

19 Dunn, L. B., Damesyn, M., Moore, A. A., Reuben, D. B. & Greendale, G. A. Does estrogen prevent skin aging? Results from the First National Health and Nutrition Examination Survey (NHANES I). *Arch Dermatol* **133**, 339-342, doi:10.1001/archderm.133.3.339 (1997).

20 Ekiz, O. *et al.* Factors influencing skin ageing in a Mediterranean population from Turkey. *Clin Exp Dermatol* **37**, 492-496, doi:10.1111/j.1365-2230.2012.04386.x (2012).

21 Elfakir, A. *et al.* Functional MC1R-gene variants are associated with increased risk for severe photoaging of facial skin. *J Invest Dermatol* **130**, 1107-1115, doi:10.1038/jid.2009.366 (2010).

22 Engel, A., Johnson, M. L. & Haynes, S. G. Health effects of sunlight exposure in the United States. Results from the first National Health and Nutrition Examination Survey, 1971-1974. *Arch Dermatol* **124**, 72-79 (1988).

23 Ernster, V. L. *et al.* Facial wrinkling in men and women, by smoking status. *Am J Public Health* **85**, 78-82, doi:10.2105/ajph.85.1.78 (1995).

24 Eun, H. C. Cutaneous photodamage in Asians. *J Dermatol* **28**, 614-616, doi:10.1111/j.1346-8138.2001.tb00045.x (2001).

25 Ezzedine, K. *et al.* Freckles and solar lentigines have different risk factors in Caucasian women. *J Eur Acad Dermatol Venereol* **27**, e345-356, doi:10.1111/j.1468-3083.2012.04685.x (2013).

26 Flament, F. & Qiu, H. Effects of seasonality and a daily photo-protection upon some facial signs of Chinese women. *Int J Cosmet Sci* **39**, 256-268, doi:10.1111/ics.12369 (2017).

27 Flament, F. *et al.* Effect of the sun on visible clinical signs of aging in Caucasian skin. *Clin Cosmet Investig Dermatol* **6**, 221-232, doi:10.2147/CCID.S44686 (2013).

28 Flament, F. *et al.* Solar exposure(s) and facial clinical signs of aging in Chinese women: impacts upon age perception. *Clin Cosmet Investig Dermatol* **8**, 75-84, doi:10.2147/CCID.S72244 (2015).

29 Flament, F., Bourokba, N., Nouveau, S., Li, J. & Charbonneau, A. A severe chronic outdoor urban pollution alters some facial aging signs in Chinese women. A tale of two cities. *Int J Cosmet Sci* **40**, 467-481, doi:10.1111/ics.12487 (2018).

30 Flament, F., Ye, C. & Amar, D. Assessing the impact of an aerial chronic urban pollution (UP) on some facial signs of differently-aged Chinese men. *Int J Cosmet Sci* **41**, 450-461, doi:10.1111/ics.12558 (2019).

31 Flament, F. *et al.* Clinical impacts of sun exposures on the faces and hands of Japanese women of different ages. *Int J Cosmet Sci* **41**, 425-436, doi:10.1111/ics.12555 (2019).

32 Flament, F., Amar, D., Forichon, M., Caron, J. & Negre, C. Distinct Habits Of Sun Exposures Lead To Different Impacts On Some Facial Signs Of Chinese Men Of Different Ages. *Clin Cosmet Investig Dermatol* **12**, 833-841, doi:10.2147/CCID.S226331 (2019).

33 Fritschi, L. & Green, A. Sun damage in teenagers' skin. *Australian Journal of Public Health* **19**, 383-386, doi:<https://doi.org/10.1111/j.1753-6405.1995.tb00390.x> (1995).

34 Fuks, K. B. *et al.* Tropospheric ozone and skin aging: Results from two German cohort studies. *Environ Int* **124**, 139-144, doi:10.1016/j.envint.2018.12.047 (2019).

35 Gao, Q. *et al.* An epidemiological survey of skin damage on the dorsal hand in rural populations in northern and southern China. *J Photochem Photobiol B* **120**, 163-170, doi:10.1016/j.jphotobiol.2012.11.010 (2013).

36 Garbe, C. *et al.* Associated factors in the prevalence of more than 50 common melanocytic nevi, atypical melanocytic nevi, and actinic lentigines: multicenter case-control study of the Central Malignant Melanoma Registry of the German Dermatological Society. *J Invest Dermatol* **102**, 700-705, doi:10.1111/1523-1747.ep12374298 (1994).

37 Gill, D., Dorevitch, A. & Marks, R. The Prevalence of Seborrheic Keratoses in People Aged 15 to 30 Years: Is the Term Senile Keratosis Redundant? *Archives of Dermatology* **136**, 759-762, doi:10.1001/archderm.136.6.759 (2000).

38 Goodman, G. J., Armour, K. S., Kolodziejczyk, J. K., Santangelo, S. & Gallagher, C. J. Comparison of self-reported signs of facial ageing among Caucasian women in Australia versus those in the USA, the UK and Canada. *Australas J Dermatol* **59**, 108-117, doi:10.1111/ajd.12637 (2018).

39 Green, A. *et al.* Skin cancer in a Queensland population. *J Am Acad Dermatol* **19**, 1045-1052, doi:10.1016/s0190-9622(88)70270-4 (1988).

40 Green, A. C., Hughes, M. C., McBride, P. & Fourtanier, A. Factors associated with premature skin aging (photoaging) before the age of 55: a population-based study. *Dermatology* **222**, 74-80, doi:10.1159/000322623 (2011).

41 Green, A. C. Premature ageing of the skin in a Queensland population. *Medical Journal of Australia* **155**, 473-478, doi:<https://doi.org/10.5694/j.1326-5377.1991.tb93845.x> (1991).

42 Gunn, D. A. *et al.* Lifestyle and youthful looks. *Br J Dermatol* **172**, 1338-1345, doi:10.1111/bjd.13646 (2015).

43 Guyuron, B. *et al.* Factors contributing to the facial aging of identical twins. *Plast Reconstr Surg* **123**, 1321-1331, doi:10.1097/PRS.0b013e31819c4d42 (2009).

44 Hamer, M. A. *et al.* Lifestyle and Physiological Factors Associated with Facial Wrinkling in Men and Women. *J Invest Dermatol* **137**, 1692-1699, doi:10.1016/j.jid.2017.04.002 (2017).

45 Helfrich, Y. R. *et al.* Effect of smoking on aging of photoprotected skin: evidence gathered using a new photonumeric scale. *Arch Dermatol* **143**, 397-402, doi:10.1001/archderm.143.3.397 (2007).

46 Hillebrand, G. G. *et al.* Quantitative evaluation of skin condition in an epidemiological survey of females living in northern versus southern Japan. *J Dermatol Sci* **27 Suppl 1**, S42-52, doi:10.1016/s0923-1811(01)00118-9 (2001).

47 Holman, C. D., Evans, P. R., Lumsden, G. J. & Armstrong, B. K. The determinants of actinic skin damage: problems of confounding among environmental and constitutional variables. *Am J Epidemiol* **120**, 414-422, doi:10.1093/oxfordjournals.aje.a113906 (1984).

48 Huls, A. *et al.* Traffic-Related Air Pollution Contributes to Development of Facial Lentigines: Further Epidemiological Evidence from Caucasians and Asians. *J Invest Dermatol* **136**, 1053-1056, doi:10.1016/j.jid.2015.12.045 (2016).

49 Huls, A., Sugiri, D., Fuks, K., Krutmann, J. & Schikowski, T. Lentigine Formation in Caucasian Women-Interaction between Particulate Matter and Solar UVR. *J Invest Dermatol* **139**, 974-976, doi:10.1016/j.jid.2018.09.034 (2019).

50 Ichibori, R. *et al.* Objective assessment of facial skin aging and the associated environmental factors in Japanese monozygotic twins. *Journal of Cosmetic Dermatology* **13**, 158-163, doi:<https://doi.org/10.1111/jocd.12081> (2014).

51 Ippen, M. & Ippen, H. Approaches to a Prophylaxis of Skin Aging. *J Soc Cosmetic Chemists* **16**, 305-308 (1965).

52 Jacobs, L. C. *et al.* Intrinsic and extrinsic risk factors for sagging eyelids. *JAMA Dermatol* **150**, 836-843, doi:10.1001/jamadermatol.2014.27 (2014).

53 Kadunce, D. P. *et al.* Cigarette smoking: risk factor for premature facial wrinkling. *Ann Intern Med* **114**, 840-844, doi:10.7326/0003-4819-114-10-840 (1991).

54 Kennedy, C. *et al.* Effect of smoking and sun on the aging skin. *J Invest Dermatol* **120**, 548-554, doi:10.1046/j.1523-1747.2003.12092.x (2003).

55 Kennedy, C., Bajdik, C. D., Willemze, R., De Gruijl, F. R. & Bouwes Bavinck, J. N. The influence of painful sunburns and lifetime sun exposure on the risk of actinic keratoses, seborrheic warts, melanocytic nevi, atypical nevi, and skin cancer. *J Invest Dermatol* **120**, 1087-1093, doi:10.1046/j.1523-1747.2003.12246.x (2003).

56 Keough, G. C., Laws, R. A. & Elston, D. M. Favre-Racouchot Syndrome: A Case for Smokers' Comedones. *Archives of Dermatology* **133**, 796-797, doi:10.1001/archderm.1997.03890420142027 (1997).

57 Kim, E. J. *et al.* Effect of the regional environment on the skin properties and the early wrinkles in young Chinese women. *Skin Res Technol* **20**, 498-502, doi:10.1111/srt.12144 (2014).

58 Kimlin, M. G. & Guo, Y. Assessing the impacts of lifetime sun exposure on skin damage and skin aging using a non-invasive method. *Sci Total Environ* **425**, 35-41, doi:10.1016/j.scitotenv.2012.02.080 (2012).

59 Knuutinen, A., Kallioinen, M., Vähäkangas, K. & Oikarinen, A. Smoking and skin: a study of the physical qualities and histology of skin in smokers and non-smokers. *Acta Derm Venereol* **82**, 36-40, doi:10.1080/000155502753600867 (2002).

60 Koh, J. S., Kang, H., Choi, S. W. & Kim, H. O. Cigarette smoking associated with premature facial wrinkling: image analysis of facial skin replicas. *Int J Dermatol* **41**, 21-27, doi:10.1046/j.1365-4362.2002.01352.x (2002).

61 Kwon, O. S. *et al.* Seborrheic keratosis in the Korean males: causative role of sunlight. *Photodermatol Photoimmunol Photomed* **19**, 73-80, doi:10.1034/j.1600-0781.2003.00025.x (2003).

62 Latreille, J. *et al.* Association between dietary intake of n-3 polyunsaturated fatty acids and severity of skin photoaging in a middle-aged Caucasian population. *J Dermatol Sci* **72**, 233-239, doi:10.1016/j.jdermsci.2013.07.006 (2013).

63 Leung, W. C. & Harvey, I. Is skin ageing in the elderly caused by sun exposure or smoking? *Br J Dermatol* **147**, 1187-1191, doi:10.1046/j.1365-2133.2002.04991.x (2002).

64 Li, M. *et al.* Epidemiological evidence that indoor air pollution from cooking with solid fuels accelerates skin aging in Chinese women. *J Dermatol Sci* **79**, 148-154, doi:10.1016/j.jdermsci.2015.04.001 (2015).

65 Lichterfeld, A., Lahmann, N., Blume-Peytavi, U. & Kottner, J. Dry skin in nursing care receivers: A multi-centre cross-sectional prevalence study in hospitals and nursing homes. *Int J Nurs Stud* **56**, 37-44, doi:10.1016/j.ijnurstu.2016.01.003 (2016).

66 Lucas, R. M. *et al.* Associations between silicone skin cast score, cumulative sun exposure, and other factors in the ausimmune study: a multicenter Australian study. *Cancer Epidemiol Biomarkers Prev* **18**, 2887-2894, doi:10.1158/1055-9965.Epi-09-0191 (2009).

67 Malvy, J.-M. *et al.* Epidemiologic determinants of skin photoaging: baseline data of the SU.VI.MAX. cohort. *J Am Acad Dermatol* **42**, 47-55, doi:10.1016/s0190-9622(00)90008-2 (2000).

68 Martires, K. J., Fu, P., Polster, A. M., Cooper, K. D. & Baron, E. D. Factors That Affect Skin Aging: A Cohort-Based Survey on Twins. *Archives of Dermatology* **145**, 1375-1379, doi:10.1001/archdermatol.2009.303 (2009).

69 Mayes, A. E. *et al.* Environmental and lifestyle factors associated with perceived facial age in Chinese women. *PLoS One* **5**, e15270, doi:10.1371/journal.pone.0015270 (2010).

70 Mekic, S. *et al.* A healthy diet in women is associated with less facial wrinkles in a large Dutch population-based cohort. *J Am Acad Dermatol* **80**, 1358-1363 e1352, doi:10.1016/j.jaad.2018.03.033 (2019).

71 Mekic, S. *et al.* Prevalence and determinants for xerosis cutis in the middle-aged and elderly population: A cross-sectional study. *J Am Acad Dermatol* **81**, 963-969 e962, doi:10.1016/j.jaad.2018.12.038 (2019).

72 Mekic, S. *et al.* Epidemiology and determinants of facial telangiectasia: a cross-sectional study. *J Eur Acad Dermatol Venereol* **34**, 821-826, doi:10.1111/jdv.15996 (2020).

73 Mizuno, M. *et al.* The effects of continuous application of sunscreen on photoaged skin in Japanese elderly people - the relationship with the usage. *Clin Cosmet Investig Dermatol* **9**, 95-105, doi:10.2147/CCID.S104392 (2016).

74 Model, D. Smoker's face: an underrated clinical sign? *British Medical Journal (Clinical research ed.)* **291**, 1760-1762, doi:10.1136/bmj.291.6511.1760 (1985).

75 Monestier, S., Gaudy, C., Gouvernet, J., Richard, M. A. & Grob, J. J. Multiple senile lentigos of the face, a skin ageing pattern resulting from a life excess of intermittent sun exposure in dark-skinned caucasians: a case-control study. *Br J Dermatol* **154**, 438-444, doi:10.1111/j.1365-2133.2005.06996.x (2006).

76 Muizzuddin, N., Marenus, K., Vallon, P. & Maes, D. Effect of cigarette smoke on skin. *Journal of the Society of Cosmetic Chemists* **48**, 235-242 (1997).

77 Nagata, C. *et al.* Association of dietary fat, vegetables and antioxidant micronutrients with skin ageing in Japanese women. *British Journal of Nutrition* **103**, 1493-1498, doi:10.1017/s0007114509993461 (2010).

78 Nouveau-Richard, S. *et al.* Skin ageing: a comparison between Chinese and European populations. A pilot study. *J Dermatol Sci* **40**, 187-193, doi:10.1016/j.jdermsci.2005.06.006 (2005).

79 O'Hare, P. M. *et al.* Tobacco smoking contributes little to facial wrinkling. *J Eur Acad Dermatol Venereol* **12**, 133-139 (1999).

80 Oyetakin-White, P. *et al.* Does poor sleep quality affect skin ageing? *Clin Exp Dermatol* **40**, 17-22, doi:10.1111/ced.12455 (2015).

81 Okada, H. C., Alleyne, B., Varghai, K., Kinder, K. & Guyuron, B. Facial changes caused by smoking: a comparison between smoking and nonsmoking identical twins. *Plast Reconstr Surg* **132**, 1085-1092, doi:10.1097/PRS.0b013e3182a4c20a (2013).

82 Park, J. H., Oh, S. J. & Lee, J. H. Effects of particulate matter on healthy human skin: a panel study using a smartphone application measuring daily skin condition. *J Eur Acad Dermatol Venereol* **33**, 1363-1368, doi:10.1111/jdv.15517 (2019).

83 Peng, F. *et al.* Exposure to fine particulate matter associated with senile lentigo in Chinese women: a cross-sectional study. *J Eur Acad Dermatol Venereol* **31**, 355-360, doi:10.1111/jdv.13834 (2017).

84 Perner, D. *et al.* Association between sun-exposure, smoking behaviour and plasma antioxidant levels with the different manifestation of skin ageing signs between Japanese and German women--a pilot study. *J Dermatol Sci* **62**, 138-140, doi:10.1016/j.jdermsci.2011.02.010 (2011).

85 Purba, M. B. *et al.* Skin wrinkling: can food make a difference? *J Am Coll Nutr* **20**, 71-80, doi:10.1080/07315724.2001.10719017 (2001).

86 Qiu, H. *et al.* Influence of season on some skin properties: winter vs. summer, as experienced by 354 Shanghaiese women of various ages. *Int J Cosmet Sci* **33**, 377-383, doi:10.1111/j.1468-2494.2011.00639.x (2011).

87 Raduan, A. P., Luiz, R. R. & Manela-Azulay, M. Association between smoking and cutaneous ageing in a Brazilian population. *J Eur Acad Dermatol Venereol* **22**, 1312-1318, doi:10.1111/j.1468-3083.2008.02804.x (2008).

88 Raitio, A. *et al.* Comparison of clinical and computerized image analyses in the assessment of skin ageing in smokers and non-smokers. *Acta Derm Venereol* **84**, 422-427, doi:10.1080/00015550410014969 (2004).

89 Rexbye, H. *et al.* Influence of environmental factors on facial ageing. *Age Ageing* **35**, 110-115, doi:10.1093/ageing/afj031 (2006).

90 Schafer, T. *et al.* The epidemiology of nevi and signs of skin aging in the adult general population: Results of the KORA-survey 2000. *J Invest Dermatol* **126**, 1490-1496, doi:10.1038/sj.jid.5700269 (2006).

91 Seddon, J. M. *et al.* Evaluation of skin microtopography as a measure of ultraviolet exposure. *Investigative Ophthalmology & Visual Science* **33**, 1903-1908 (1992).

92 Silva Idos, S. *et al.* Overseas sun exposure, nevus counts, and premature skin aging in young English women: a population-based survey. *J Invest Dermatol* **129**, 50-59, doi:10.1038/jid.2008.190 (2009).

93 Singer, R. S., Hamilton, T. A., Voorhees, J. J. & Griffiths, C. E. Association of asymmetrical facial photodamage with automobile driving. *Arch Dermatol* **130**, 121-123, doi:10.1001/archderm.1994.01690010127031 (1994).

94 Song, E. J. *et al.* A study on seasonal variation of skin parameters in Korean males. *Int J Cosmet Sci* **37**, 92-97, doi:10.1111/ics.12174 (2015).

95 Suehara, L. Y., Simone, K. & Maia, M. Evaluation of facial aging related to cigarette smoking. *Anais Brasileiros de Dermatologia* **81**, 34-39 (2006).

96 Suppa, M. *et al.* The determinants of periorbital skin ageing in participants of a melanoma case-control study in the U.K. *Br J Dermatol* **165**, 1011-1021, doi:10.1111/j.1365-2133.2011.10536.x (2011).

97 Takahashi, Y., Fukushima, Y., Kondo, K. & Ichihashi, M. Facial skin photo-aging and development of hyperpigmented spots from children to middle-aged Japanese woman. *Skin Res Technol* **23**, 613-618, doi:10.1111/srt.12380 (2017).

98 Tsukahara, K. *et al.* Comparison of age-related changes in facial wrinkles and sagging in the skin of Japanese, Chinese and Thai women. *J Dermatol Sci* **47**, 19-28, doi:10.1016/j.jdermsci.2007.03.007 (2007).

99 Tsukahara, K. *et al.* Gender-dependent differences in degree of facial wrinkles. *Skin Res Technol* **19**, e65-71, doi:10.1111/j.1600-0846.2011.00609.x (2013).

100 Tsukahara, K., Osanai, O., Kitahara, T. & Takema, Y. Seasonal and annual variation in the intensity of facial wrinkles. *Skin Res Technol* **19**, 279-287, doi:10.1111/srt.12038 (2013).

101 Vierkotter, A. *et al.* The SCINEXA: a novel, validated score to simultaneously assess and differentiate between intrinsic and extrinsic skin ageing. *J Dermatol Sci* **53**, 207-211, doi:10.1016/j.jdermsci.2008.10.001 (2009).

102 Vierkotter, A. *et al.* Airborne particle exposure and extrinsic skin aging. *J Invest Dermatol* **130**, 2719-2726, doi:10.1038/jid.2010.204 (2010).

103 Vierkotter, A. *et al.* Extrinsic skin ageing in German, Chinese and Japanese women manifests differently in all three groups depending on ethnic background, age and anatomical site. *J Dermatol Sci* **83**, 219-225, doi:10.1016/j.jdermsci.2016.05.011 (2016).

104 Wang, Y. N., Fang, H. & Zhu, W. F. Survey on skin aging status and related influential factors in Southeast China. *J Zhejiang Univ Sci B* **10**, 57-66, doi:10.1631/jzus.B0820071 (2009).

105 Warren, R. *et al.* Age, sunlight, and facial skin: a histologic and quantitative study. *J Am Acad Dermatol* **25**, 751-760, doi:10.1016/s0190-9622(08)80964-4 (1991).

106 Yin, L., Morita, A. & Tsuji, T. Skin aging induced by ultraviolet exposure and tobacco smoking: evidence from epidemiological and molecular studies. *Photodermatol Photoimmunol Photomed* **17**, 178-183, doi:10.1034/j.1600-0781.2001.170407.x (2001).

107 Yin, L., Morita, A. & Tsuji, T. Skin premature aging induced by tobacco smoking: the objective evidence of skin replica analysis. *J Dermatol Sci* **27 Suppl 1**, S26-31, doi:10.1016/s0923-1811(01)00112-8 (2001).

108 Youn, C. S. *et al.* Effect of pregnancy and menopause on facial wrinkling in women. *Acta Derm Venereol* **83**, 419-424, doi:10.1080/00015550310015464 (2003).

109 Zhao, P. *et al.* Solar ultraviolet radiation and skin damage: an epidemiological study among a Chinese population. *Arch Environ Health* **53**, 405-409, doi:10.1080/00039899809605728 (1998).
